# Membrane-Active Thermoresponsive Block Copolymers Containing a Diacylglycerol-Based Segment: RAFT Synthesis, Doxorubicin Encapsulation, and Evaluation of Cytotoxicity against Breast Cancer Cells

**DOI:** 10.1021/acs.biomac.3c00580

**Published:** 2023-10-16

**Authors:** Izabela Kurowska, Karolina H. Markiewicz, Katarzyna Niemirowicz-Laskowska, Mathias Destarac, Przemysław Wielgat, Iwona Misztalewska-Turkowicz, Paweł Misiak, Halina Car, Agnieszka Z. Wilczewska

**Affiliations:** † Faculty of Chemistry, University of Bialystok, Ciolkowskiego 1K, Bialystok 15-245, Poland; ‡ Doctoral School of Exact and Natural Sciences, University of Bialystok, Bialystok 15-245, Poland; § Department of Experimental Pharmacology, Medical University of Bialystok, Szpitalna 37, Bialystok 15-295, Poland; ∥ Laboratoire IMRCP, CNRS UMR 5623, Paul Sabatier University, Toulouse Cedex 09 31062, France; ⊥ Department of Clinical Pharmacology, Medical University of Bialystok, Waszyngtona 15A, Bialystok 15-274, Poland

## Abstract

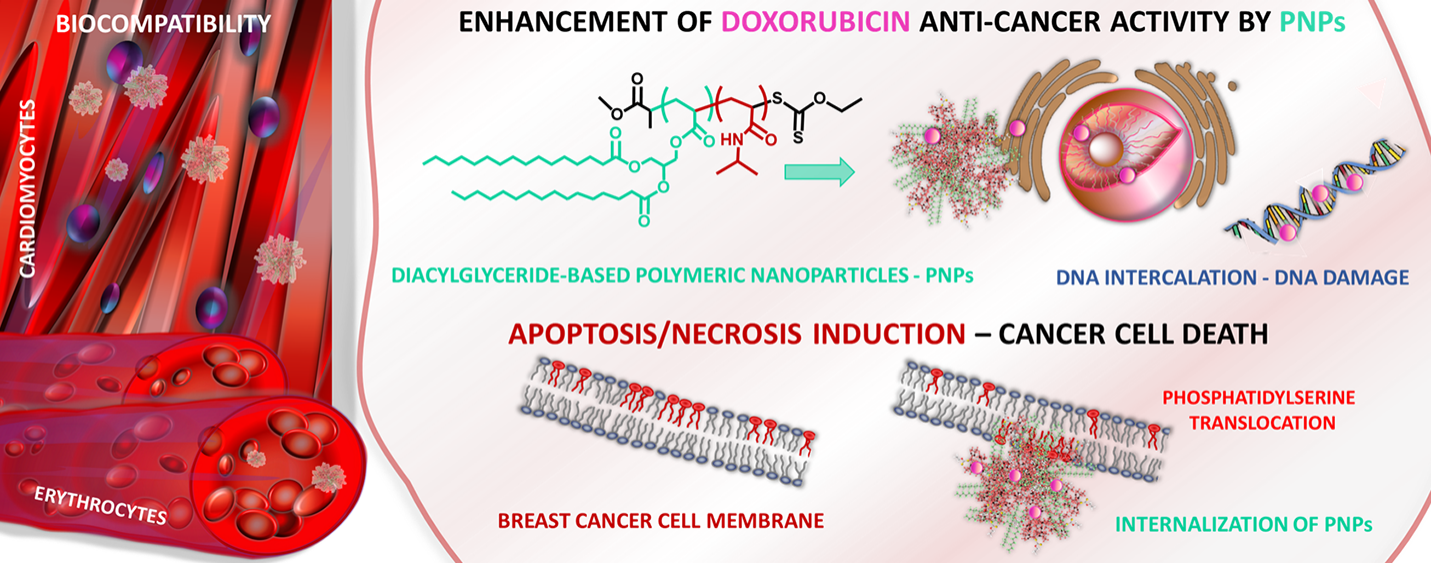

Herein, we report the formation of drug delivery systems from original thermoresponsive block copolymers containing lipid-based segments. Two acrylate monomers derived from palmitic- or oleic-acid–based diacylglycerols (DAGs) were synthesized and polymerized by the reversible addition–fragmentation chain transfer (RAFT) method. Well-defined DAG-based polymers with targeted molar masses and narrow molar mass distributions were next used as macro-chain transfer agents (macro-CTAs) for the polymerization of *N*-isopropylacrylamide (NIPAAm) or *N*-vinylcaprolactam (NVCL). The obtained amphiphilic block copolymers were formed into polymeric nanoparticles (PNPs) with and without encapsulated doxorubicin and characterized. Their biological assessment indicated appropriate cytocompatibility with the representatives of normal cells. Furthermore, compared to the free drug, increased cytotoxicity and apoptosis or necrosis induction in breast cancer cells was documented, including a highly aggressive and invasive triple-negative MDA-MB-231 cell line.

## 1 Introduction

In recent years, numerous polymeric nanosized drug delivery systems (DDS) have been developed to improve therapeutic efficacy and reduce side effects of active agents.^1,2^ It is due to an impressive advance in synthetic methodologies, mainly controlled radical polymerization, and an understanding of the physicochemical behavior of polymers made in the last few decades. Well-defined macromolecules combining a unique architecture with several functions in one chemical entity can be easily obtained. This might be used to obtain nanocarriers with tailor-made properties such as high drug encapsulation efficiency, excellent stability providing sustained drug delivery, and stimuli-responsiveness enabling control over bioactive molecule release. However, mainly due to the poor membrane permeability, polymeric nanocarriers present low drug delivery efficiency.^3^ One strategy to address this issue is the incorporation of membrane-active mediators into macromolecular DDS.^4^ Lipids, due to their biomimetic nature, might promote interactions with the plasma membrane and improve cellular uptake of the drug-carrier system.^5^ In this view, the synthesis of lipid-polymer conjugates (LPCs) might lead to stable and biocompatible nanocarriers showing high affinity to cell membranes, enabling encapsulation, delivery, and controlled release of bioactive molecules with diverse activity and mode of action that cannot be applied effectively through conventional routes.^3^

In the last decades, the knowledge about the structure and functions of biological membranes has expanded.^6^ Among a plethora of bioactive lipids present in biological membranes, diacylglycerols (diglycerides, DAGs, or DGs) are quantitatively minor but functionally significant components. DAGs help maintain membrane homeostasis, participating in diverse metabolic processes and signaling pathways.^7,8^ Moreover, the amount of DAGs influences the physical and chemical characteristics of the membrane, such as its curvature or how it interacts with proteins.^8−10^ Diacylglycerols can exist in various stereochemical forms and may incorporate diverse fatty acid (FA) moieties esterified to the glycerol backbone, which significantly influences their biological properties.^11,12^ Regarding plasma membranes, saturated fatty acids render the structure more ordered and rigid. In contrast, unsaturated acids, which do not pack as tightly, promote some fluidity in the membrane.

Lipid–polymer conjugates might contain DAGs in the main chain or as side chains. So far, only several DAG end-functionalized polymers have been reported.^13−15^ Diglyceride-ended poly(oligo(ethylene glycol)acrylates)^13^ and acrylamides^14^ were obtained by reversible addition–fragmentation chain-transfer (RAFT) polymerizations mediated by DAG-based trithiocarbonates. Recently, such an approach was also used in our group to prepare DAG-terminated poly(*N*-isopropylacrylamide)s (PNIPAAm) and poly(*N*-vinylcaprolactam)s (PNVCL) with a controlled structure. We used two dithiocarbonate-functionalized DAGs derived from palmitic or oleic acid as chain transfer agents (CTAs).^15^ The obtained LPCs were further formed into nanoparticles (NPs) with well-defined size and high stability in water and PBS. The phase transition temperature of such NPs depends on polymer chain length and hydrophilic–lipophilic balance. Biological studies showed that the nanoparticles were compatible with red blood cells and selected immune cells. However, no significant differences in terms of biological properties, depending on the molar mass and saturation of the fatty acids used, were observed. Considering the promising biological results and structure–activity relationship investigation, we decided to expand our knowledge and study LPCs containing diglycerides as side chains. The results, which demonstrate that the multiple long alkyl side chains, acting together in the polymer, induce the disruption of the membrane and release of cytoplasmic contents, ultimately causing the microorganisms’ death,^16^ additionally encouraged our further research.

Therefore, herein, we report the formation of a drug delivery system containing thermoresponsive and diacylglycerol-based blocks. First, two original acrylate monomers based on diacylglycerols bearing palmitic or oleic acid moieties were synthesized and polymerized by the RAFT method. Next, copolymerizations with either *N*-isopropylacrylamide (NIPAAm) or *N*-vinylcaprolactam (NVCL) were performed. The incorporation of PNVCL or PNIPAAm as a hydrophilic block attached to the diglyceride block gives amphiphilic character and self-assembly properties to the copolymers, which allow for the encapsulation of DOX in well-defined nanosized containers. Additionally, we decided to take advantage of the phase transition property of hydrophilic PNIPAM and PNVCL with LCST temperatures below the body temperature. We assumed that at body temperature, the hydrophilic PNIPAM or PNVCL corona of the DDS should be in a dehydrated state, with an increased hydrophobic character promoting interactions with the cancer cell membrane for better disruption by the lipid block. The four obtained well-defined amphiphilic block copolymers were formed into polymeric nanoparticles (NPs) with and without encapsulated doxorubicin and characterized in terms of their physicochemical properties. Eventually, an in-depth biological evaluation of the obtained systems was performed. The cytocompatibility was tested with selected normal cells, such as human erythrocyte, monocytic, and cardiomyocyte cells. Cytotoxic activity, internalization, and apoptosis/necrosis evaluation against estrogen-dependent and estrogen-independent breast cancer cells, including an aggressive and invasive triple-negative MDA-MB-231 cell line, was investigated.

## 2 Materials and Methods

### 2.1 Materials

Solketal (97%, Alfa Aesar), sodium hydride (60% dispersion in mineral oil, Sigma-Aldrich), benzyl bromide (99%, Alfa Aesar), palmitic acid (99%, Sigma-Aldrich), oleic acid (90%, Sigma-Aldrich), *N,N′*-dicyclohexylcarbodiimide (DCC, 99%, Sigma-Aldrich), 4-dimethylaminopyridine (DMAP, 99%, Aldrich), boron trichloride solution (BCl_3_, 1.0 M in methylene chloride, Sigma-Aldrich), palladium on activated charcoal (Pd/C, 5% Pd basis, Sigma-Aldrich), acryloyl chloride (96%, Alfa Aesar), triethylamine (Et_3_N, Avantor), fluorescein diacetate 5-maleimide (Sigma-Aldrich), *n*-propylamine (PROSYNTH), tributylphosphine (97%, Sigma-Aldrich), phosphate buffer saline (PBS, pH = 7.4, Gibco), and doxorubicin hydrochloride (DOX, AmBeed) were used as received. *N*-Isopropylacrylamide (NIPAAm, 99%, Acros) was recrystallized from toluene–hexane (60:40, v/v).^17^*N*-Vinylcaprolactam (NVCL) was recrystallized three times from hexane at room temperature. 2,2′-Azobis(2-methylpropionitrile) (AIBN, 98%, Merck) was recrystallized from methanol.^18^ Dialysis membrane with 3.5 kDa MWCO (molecular weight cutoff) was purchased from Spectra/Por. Methyl 2-((ethoxycarbonothioyl)thio)propanoate (CTA1),^18^ PNIPAAm (*M*_n_ = 10160 g·mol^–1^, *Đ* = 1.13),^17^ and PNVCL (*M*_n_ = 7600 g·mol^–1^, *Đ* = 1.6)^15^ were synthesized following previously established methods. All organic solvents were purchased from Avantor Performance Materials, Poland S.A., and distilled before use.

### 2.2 Experimental Section

#### 2.2.1 Synthesis of 3-(Benzyloxy)propane-1,2-diol (**1**)

Sodium hydride (60 wt % in mineral oil, 3.3 g, 1.3 equiv) was washed with hexane (3 × 20 mL) and then dissolved in THF (200 mL). Next, solketal (9.4 mL, 7.65 mmol) was introduced at 0 °C, and the resulting mixture was stirred for 1 h. Subsequently, benzyl bromide (10.83 mL, 0.09 mol, 1.2 equiv) was added portionwise. The reaction mixture was warmed to room temperature and stirred overnight. The reaction mixture was then treated with 150 mL of a saturated ammonium chloride (NH_4_Cl) solution, and the product was extracted with diethyl ether (3 × 150 mL). The combined organic layer was dried over anhydrous sodium sulfate (Na_2_SO_4_), filtered, and concentrated under reduced pressure. The remaining residue was dissolved in an acetic acid/water (AcOH/H_2_O) solution with a 4:1 ratio (100 mL). The mixture was stirred at 65 °C for 3 h and neutralized with a saturated aqueous sodium bicarbonate (NaHCO_3_) solution (150 mL). The product was isolated by extraction with dichloromethane (DCM) (3 × 150 mL). The organic layer was dried over Na_2_SO_4_, filtered, and concentrated under reduced pressure. The residue was purified by medium-pressure liquid chromatography (MPLC) with hexane–ethyl acetate (EtOAc) (6.5:3.5 and 0:10). The pale yellow oil was obtained with an efficiency of 71%, 9.703 g.

^1^H NMR (400 MHz, CDCl_3_) δ 7.51–7.17 (m, 5H, C_6_***H*_5_**), 4.53 (s, 2H, C***H*_2_**C_6_H_5_), 3.95–3.80 (m, 1H, COCH_2_C***H***CH_2_), 3.66 (dd, *J* = 11.5, 3.6 Hz, 2H, HOC***H***_**2**_CH), 3.61–3.52 (m, 2H, CHC***H***_**2**_O), 3.50 (dd, *J* = 5.2, 3.3 Hz, 2H, CHC***H***_**2**_O).

^13^C NMR (101 MHz, CDCl_3_): δ 137.6 (C), 128.4 (CH), 127.7 (CH), 73.4 (CH_2_), 71.5 (CH_2_), 70.7 (CH), 63.9 (CH_2_).

FT-IR (ATR, cm^–1^): ν 3335 (O–H), 3065, 3030, 2925, 2870 (C–H), 1715, 1450, 1275, 1095, 1055, 740, 700.

#### 2.2.2 Synthesis of 3-(Benzyloxy)propane-1,2-diyl Dipalmitate (**2a**)

Compound **1** (2.5 g, 13.73 mmol) and DMAP (0.67 g, 5.5 mmol, 0.4 equiv) were dissolved in 200 mL of dry DCM. Next, DCC (7.08 g, 34.6 mmol, 2.5 equiv) was added portionwise at 0 °C. After 1 h of stirring, palmitic acid (3.74 g, 16.6 mmol, 2.2 equiv) was added gradually. The reaction mixture was warmed to room temperature and stirred overnight. The solid side product, dicyclohexylurea (DCU), was separated by passing the mixture through Celite, and the excess solvent was removed by distillation under reduced pressure. The residue was subjected to MPLC chromatography (hexane–EtOAc = 9.5:0.5). The white solid product was obtained with an efficiency of 90%, 8.16 g.

^1^H NMR (400 MHz, CDCl_3_): δ 7.38–7.26 (m, 5 H, C_6_***H*_5_**), 5.29–5.22 (m, 1 H, COCH_2_C***H***CH_2_), 4.54 (d, *J* = 5.6 Hz, 2 H, C***H***_**2**_C_6_H_5_), 4.36 (dd, *J* = 11.9, 3.8 Hz, 2 H, CHC***H***_**2**_C(=O)), 4.20 (dd, *J* = 11.9 Hz, 6.4 Hz, 2 H, CHC***H*_2_**C(=O)), 3.60 (m, 2 H, CHC***H***_**2**_OCH_2_), 2.31 (dt, *J* = 17.0, 7.5 Hz, 4H, C***H*_2_**C(=O)), 1.70–1.53 (m, 4 H, C***H***_**2**_CH_2_C(=O)), 1.28 (s, 48 H, C***H*_2_**), 0.89 (t, *J* = 6.8 Hz, 6 H, C***H***_**3**_).

^13^C NMR (101 MHz, CDCl_3_): δ 173.4 (C=O), 173.1 (C=O), 137.7 (C), 128.4 (CH), 127.8 (CH), 127.60 (CH), 73.3 (CH_2_), 70.0 (CH), 68.2 (CH_2_), 62.6 (CH_2_), 34.3 (CH_2_), 34.1(CH_2_), 31.9 (CH_2_), 29.7 (CH_2_), 29.6 (CH_2_), 29.5 (CH_2_), 29.4 (CH_2_), 29.3 (CH_2_), 29.1 (CH_2_), 29.0 (CH_2_), 25.0 (CH_2_), 24.9 (CH_2_), 22.7 (CH_2_), 14.1 (CH_3_).

FT-IR (ATR, cm^–1^): ν 2955 (C–H), 2920 (C–H), 2850 (C–H), 1740, 1720, 1450, 1375, 1350, 1240, 1220, 1150, 1120, 1090, 730.

#### 2.2.3 Synthesis of 3-(Benzyloxy)propane-1,2-diyl Dioleate (**2b**)

Compound **1** (2.5 g, 13.2 mmol) and DMAP (0.671 g, 5.5 mmol, 0.4 equiv) were dissolved in 200 mL of dry DCM. Next, DCC (6.8 g, 32.9 mmol, 2.5 equiv) was added portionwise at 0 °C. After 1 h of stirring, oleic acid (9.98 mL, 31.6 mmol, 2.2 equiv) was added gradually. The reaction mixture was warmed to room temperature and stirred overnight. The solid side product (dicyclohexylurea) was separated by passing the mixture through Celite, and the excess solvent was removed by distillation under reduced pressure. The residue was subjected to MPLC chromatography (hexane–EtOAc = 9.7:0.3). The pale yellow oil was obtained at 85% yield, 8.30 g.

^1^H NMR (400 MHz, CDCl_3_): δ 7.42–7.22 (m, 5H, C_6_***H*_5_**), 5.45–5.30 (m, 4H, OCH_2_C*H*=C***H***CH_2_), 5.26 (m, 1H, COC*H*_2_C***H***CH_2_), 4.55 (d, *J* = 5.5 Hz, 2H, C***H***_**2**_C_6_H_5_), 4.36 (dd, *J* = 11.9, 3.8 Hz, 2H, CHC***H*_2_**C(=O)), 4.20 (dd, *J* = 11.9, 6.4 Hz, 2H, CHC***H***_**2**_C(=O)), 3.60 (d, *J* = 5.2 Hz, 2H, CHC***H***_**2**_OCH_2_), 2.31 (dt, *J* = 17.1, 7.5 Hz, 4H, C***H*_2_**C(=O)), 2.14–1.93 (m, 8H, C***H*_2_**CH=CHC***H*_2_**), 1.68–1.53 (m, 4H, C***H***_**2**_CH_2_C(=O)), 1.31 (s, 20H, C***H***_**2**_), 1.28 (s, 20H, *C****H***_**2**_), 0.90 (t, *J* = 6.8 Hz, 6H, C***H*_3_**).

^13^C NMR (101 MHz, CDCl_3_) δ 173.3 (C=O), 173.0 (C=O), 137.7 (C), 130.0 (CH), 129.7 (CH), 128.4 (CH), 127.7 (CH), 127.6 (CH), 73.2 (CH_2_), 70.0 (CH), 68.2 (CH_2_), 62.6 (CH_2_), 34.2 (CH_2_), 34.0 (CH_2_), 31.9 (CH_2_), 29.7 (CH_2_), 29.5 (CH_2_), 29.3 (CH_2_), 29.2 (CH_2_), 29.1 (CH_2_), 29.0 (CH_2_), 27.2 (CH_2_), 27.1 (CH_2_), 24.9 (CH_2_), 24.8 (CH_2_), 22.6 (CH_2_), 14.1 (CH_3_).

FT-IR (ATR, cm^–1^): ν 3004 (C–H), 2920 (C–H), 2850 (C–H), 1740, 1455, 1365, 1350, 1240, 1160, 1095, 730, 700.

#### 2.2.4 Synthesis of 3-Hydroxypropane-1,2-diyl Dipalmitate (**3a**)

Compound **2a** (6.0 g, 10.0 mmol) and 5% Pd/C (800 mg) were dissolved in a mixture of glacial acetic acid (20 mL) and ethanol (100 mL). The resulting solution was stirred under a hydrogen atmosphere at room temperature. Upon completion of the reaction (determined by TLC), DCM was added to dilute the mixture, and the catalyst was separated by passing the mixture through Celite. The excess solvent was removed by distillation under reduced pressure, and the residue was subjected to MPLC chromatography (hexane–EtOAc = 9.6:0.4). The white solid product was obtained with an efficiency of 86%, 4.45 g.

^1^H NMR (400 MHz, CDCl_3_) δ 5.13–5.02 (m, 1H, COCH_2_C***H***CH_2_), 4.32 (dd, *J* = 11.9, 4.3 Hz, 2H, CHC***H*_2_**C(=O)), 4.24 (dd, *J* = 11.6, 5.6 Hz, CHC***H*_2_**C(=O)), 2 H, 3.79–3.63 (m, 2H, CHC***H*_2_**OH), 2.35 (td, *J* = 12.0, 5.9 Hz, 4 H, CH_2_C***H*_2_**C(=O)), 1.66–1.58 (m, 4H, C***H*_2_**CH_2_C(=O)), 1.25 (s, 48H, C***H*_2_**), 0.88 (t, *J* = 6.8 Hz, 6H, C***H*_3_**).

^13^C NMR (101 MHz, CDCl_3_): δ 173.9 (C=O), 68.3 (CH), 65.0 (CH_2_), 34.1 (CH_2_), 29.7 (CH_2_), 29.6 (CH_2_), 29.5 (CH_2_), 29.4 (CH_2_), 29.3 (CH_2_), 29.2 (CH_2_), 29.1 (CH_2_), 24.9 (CH_2_), 22.6 (CH_2_), 14.1 (CH_3_).

FT-IR (ATR, cm^–1^): ν 3500 (OH), 2955 (C–H), 2920 (C–H), 2850, 1730, 1710, 1470, 1380, 1290, 1265, 1220, 1180, 1090, 1065, 720.

#### 2.2.5 Synthesis of 3-Hydroxypropane-1,2-diyl Dioleate (**3b**)

To a solution of **2b** (1.5 g, 0.0021 mol, 1 equiv) in 50 mL of dry DCM at −78 °C was added boron trichloride (5.3 mL, 0.053 mol, 2.5 equiv) (1 M in DCM) over 15 min. The resulting mixture was stirred for 1 h under an argon atmosphere. Then, the flask content was poured over ice water (50 mL) and warmed to ambient temperature. The product was extracted with DCM (3 × 50 mL), and the combined organic layer was dried over anhydrous Na_2_SO_4_, filtered, and concentrated under reduced pressure. The residue was subjected to MPLC chromatography (hexane–EtOAc = 9.7:0.3). The pale yellow oil was obtained with an efficiency of 54%, 712 mg.

^1^H NMR (400 MHz, CDCl_3_) δ 5.46–5.21 (m, 5H, CH_2_C***H***=C***H***CH_2_, COC*H*_2_C***H***CH_2_), 4.29–4.00 (m, 4H, CHC***H*_2_**C(=O), CHC***H*_2_**OH), 2.35 (td, *J* = 7.6, 2.5 Hz, 4H, C***H***_2_C(=O))), 2.04–1.99 (m, 8H, C***H*_2_**CH = CHC***H*_2_**, 1.65–1.61 (m, 4H, C***H***_**2**_CH_2_C(=O)), 1.31 (s, 20H, C***H***_**2**_), 1.28 (s, 20H, C***H***_**2**_), 0.90 (t, *J* = 6.8 Hz, 6H, C***H*_3_**).

^13^C NMR (101 MHz, CDCl_3_) δ 173.8 (C=O), 130.0 (CH), 129.7 (CH), 68.3 (CH), 65.0 (CH_2_), 34.0 (CH_2_), 31.9 (CH_2_), 29.7 (CH_2_), 29.6 (CH_2_), 29.5 (CH_2_), 29.3 (CH_2_), 29.1 (CH_2_), 29.0 (CH_2_), 27.2 (CH_2_), 27.1 (CH_2_), 22.6 (CH_2_), 14.1 (CH_3_).

FT-IR (ATR, cm^–1^): ν 3450 (OH), 3004 (C–H), 2920, 2850, 1740, 1455, 1370, 1240, 1065, 720, 700.

#### 2.2.6 Synthesis of 3-(Acryloyloxy)propane-1,2-diyl Dipalmitate (**4a**)

Compound **3a** (4.3 g, 7.5 mmol) was dissolved in dry DCM (200 mL), and triethylamine (2 mL, 15 mmol, 2 equiv) was introduced. After 30 min, acryloyl chloride (0.75 mL, 9 mmol, 1.2 equiv) was added dropwise over 30 min at 0 °C. The reaction mixture was sustained at 0 °C for 3 h and then stirred at room temperature overnight. Next, 150 mL of a 1 M HCl solution was added, and the product was extracted with DCM (3 × 100 mL). The combined organic layer was washed with brine, dried over Na_2_SO_4,_ filtered, and concentrated under reduced pressure. The residue was purified by MPLC (hexane–EtOAc = 9.6:0.4). The white solid product was obtained with an efficiency of 89%, 5.3 g.

^1^H NMR (400 MHz, CDCl_3_) δ 6.43 (d, *J* = 17.3 Hz, 1H, C***H*_2_**=C***H*_2_**), 6.13 (dd, *J* = 17.3, 10.4 Hz, 1H, C***H*_2_**=C***H*_2_**), 5.88 (d, *J* = 10.5 Hz, 1H, C***H*_2_**=C***H*_2_**), 5.58–5.03 (m, 1H, COCH_2_C***H***CH_2_), 4.47–4.23 (m, 2H, CHC***H*_2_**C(=O)), 4.17 (dd, *J* = 11.9, 5.9 Hz, 2H, CHC***H*_2_**C(=O)), 2.32 (td, *J* = 7.5, 2.6 Hz, 4H, C***H*_2_**C(=O)), 1.63–1.59 (m, 4H, C***H*_2_**CH_2_C(=O)), 1.25 (s, 48H, C***H*_2_**), 0.89 (t, *J* = 6.6 Hz, 6H, C***H*_3_**).

^13^C NMR (101 MHz, CDCl_3_): δ 173.2 (C=O), 172.8 (C=O), 165.5 (C=O), 131.5 (CH_2_), 127.7 (CH), 68.8 (CH), 62.4 (CH_2_), 62.0 (CH_2_), 34.2 (CH_2_), 34.0 (CH_2_), 31.9 (CH_2_), 29.7 (CH_2_)_,_ 29.6 (CH_2_), 29.5 (CH_2_), 29.4 (CH_2_), 29.3 (CH_2_), 29.2 (CH_2_), 29.1 (CH_2_), 29.0 (CH_2_), 24.9 (CH_2_), 24.8 (CH_2_), 22.7 (CH_2_), 14.1 (CH_3_).

FT-IR (ATR, cm^–1^): ν 2955, 2920, 2850, 1740, 1720, 1635 (C=O), 1460, 1360, 1260, 1170, 1085, 810.

MS (ESI): *m*/*z* calculated 645.5000 (M + Na)^+^, found 645.5101.

#### 2.2.7 Synthesis of 3-(Acryloyloxy)propane-1,2-diyl Dioleate (**4b**)

Compound **3b** (2.5 g, 4 mmol) was dissolved in dry DCM (85 mL), and then, triethylamine (1.12 mL, 8 mmol, 2 equiv) was introduced. After 30 min, acryloyl chloride (0.43 mL, 5.2 mmol, 1.3 equiv) was added dropwise over 30 min at 0 °C. The reaction mixture was sustained at 0 °C for 3 h and then stirred at room temperature overnight. Next, 100 mL of a 1 M HCl solution was added, and the product was extracted with DCM (3 × 80 mL). The combined organic layer was washed with brine, dried over Na_2_SO_4,_ filtered, and concentrated under reduced pressure. The residue was purified by MPLC (hexane–EtOAc = 9.6:0.4). The yellow oil was obtained in 49% yield, 1.3 g.

^1^H NMR (400 MHz, CDCl_3_) δ 6.43 (d, *J* = 17.3, 1H, C***H*_2_**=C***H*_2_**), 6.12 (dd, *J* = 17.3, 10.4 Hz, 1H, C***H*_2_**=C***H*_2_**)), 5.87 (d, *J* = 10.5 Hz, 1H, C***H*_2_**=C***H*_2_**), 5.43–5.25 (m, 5H, CH_2_C***H***=C***H***CH_2_, COC*H*_2_C***H***CH_2_), 4.41–4.11 (m, 2H, CHC***H*_2_**C(=O)), 2.32 (td, *J* = 7.6, 2.5 Hz, 4H, C***H*_2_**C(=O)), 2.09–1.89 (m, 8H, m, 8H, C***H*_2_**CH=CHC***H*_2_**), 1.72–1.52 (m, 4H, C***H***_**2**_CH_2_C(=O)), 1.31 (s, 20H, C***H***_**2**_), 1.28 (s, 20H, C***H***_**2**_), 0.90 (t, *J* = 6.8 Hz, 6H, C***H*_3_**).

^**13**^**C NMR** (101 MHz, CDCl_3_) δ 173.2 (C=O), 172.8 (C=O), 165.5 (C=O), 131.6 (CH_2_), 130.0 (CH), 129.7 (CH), 129.7 (CH), 127.7 (CH), 68.8 (CH), 62.4 (CH_2_), 62.0 (CH_2_), 34.2 (CH_2_), 34.0 (CH_2_), 31.9 (CH_2_), 29.7 (CH_2_), 29.5 (CH_2_), 29.3 (CH_2_), 29.1 (CH_2_), 29.0 (CH_2_), 27.2 (CH_2_) 27.1 (CH_2_), 24.8 (CH_2_), 22.6 (CH_2_), 14.1 (CH_3_).

FT-IR (ATR, cm^–1^): ν 3004 (C–H), 2915 (C–H), 2850 (C–H), 1740 (C=O), 1635 (C=O), 1465, 1405, 1300, 1170, 1095, 985.

MS (ESI): *m*/*z* calculated 697.5392 (M + Na)^+^, found 697.5397.

### 2.3 General Procedure for RAFT Polymerization of GlyP Acrylate

Predetermined quantities of CTA1, GlyP-A, and AIBN were added to a Schlenk tube and dissolved in THF (50 wt %). The solution was degassed by purging with argon for 30 min and placed in an oil bath preheated to 70 °C. After 6 h, the polymerization was stopped by cooling in an ice bath, and THF was removed under reduced pressure. The polymer was purified by four cycles of solubilization in chloroform and precipitation in cold methanol, followed by filtration and drying.

### 2.4 General Procedure for RAFT Polymerization of GlyO Acrylate

Predetermined quantities of CTA1, GlyO-A, and AIBN were added to a Schlenk tube and dissolved in THF (50 wt %). The solution was degassed by purging with argon for 30 min and placed in an oil bath preheated to 70 °C. After 16 h, the polymerization was stopped by cooling in an ice bath, and THF was removed under reduced pressure. The polymer was purified by four cycles of solubilization in acetone and precipitation in cold methanol, followed by filtration and drying.

### 2.5 General Procedure for RAFT Copolymerization with NIPAAm

Predetermined quantities of GlyP or GlyO macro-CTA, NIPAAm, and AIBN were added to a Schlenk tube and dissolved in THF (50 wt %). The solution was degassed by purging with argon for 30 min and placed in an oil bath preheated to 70 °C. After 6 h, the polymerization was stopped by cooling in an ice bath, and THF was removed under reduced pressure. The polymer was purified by two cycles of solubilization in chloroform and precipitation in cold hexane, followed by filtration and drying.

### 2.6 General Procedure for RAFT Copolymerization with NVCL

Predetermined quantities of GlyP or GlyO macro-CTA, NVCL, and AIBN were added to a Schlenk tube and dissolved in THF (50 wt %). The solution was degassed by purging with argon for 30 min and placed in an oil bath preheated to 70 °C. After 16 h, the polymerization was stopped by cooling in an ice bath, and THF was removed under reduced pressure. The polymer was purified by two cycles of solubilization in chloroform and precipitation in cold pentane, followed by filtration and drying.

### 2.7 General Procedure for Polymeric Nanoparticle Formation

Polymeric nanoparticles were prepared according to a previously described method.^15,19^ The obtained polymeric nanoparticles without drugs were marked as NP. Drug-encapsulated polymeric nanoparticles were formed in a similar manner. The 10 mg of the polymer was dissolved in 1 mL of doxorubicin hydrochloride (DOX) solution in THF (*C* = mg·mL^–1^). After 3 h, the solution was added dropwise to 10 mL of distilled water with constant stirring. Next, the resulting mixture was dialyzed against 1 L of distilled water for 24 h, with the water changed twice (after 1 and 3 h). Finally, the membrane content was freeze-dried and further analyzed. The obtained polymeric nanoparticles were marked as NPDOX.

### 2.8 Methods

#### 2.8.1 Attenuated Total Reflectance Fourier Transform Infrared Spectroscopy (ATR-FTIR)

A Thermo Scientific Nicolet 6700 FTIR apparatus equipped with a diamond ATR was used to record the ATR-FTIR spectra. Spectra were collected in the wavenumber range from 4000 to 500 cm^–1^ by coadding 32 scans with a resolution of 4 cm^–1^.

#### 2.8.2 Differential Scanning Calorimetry (DSC)

Samples (2–3 mg) placed in sealed aluminum crucibles were heated from 25 to 200 °C (10 °C·min^–1^), held isothermally for 5 min, and then cooled to −100 °C (−20 °C·min^–1^) in a Mettler Toledo Star DSC unit. Two heating/cooling cycles under an argon flow rate of 200 mL·min^–1^ were performed by using an empty pan as a reference. The glass transition temperature (*T*_g_) was identified as the midpoint of the change in heat capacity in the second heating run.

#### 2.8.3 Dynamic Light Scattering (DLS)

DLS measurements were taken on a Zetasizer Ultra (Malvern Instruments, UK) equipped with a He–Ne laser of wavelength 633 nm. To determine the size, nanoparticles were dispersed in either PBS or water, ensuring a concentration of 0.5 mg·mL^–1^. Phase separation of polymeric nanoparticles was evaluated in PBS at the same concentration. Measurements were conducted in the temperature gradient from 25 to 40 °C with increments of 0.5 °C. The aggregation temperature (*T*_agg_) was determined from size versus temperature plots and was identified as an inflection point of the curve. Zeta potential was measured using a high-concentration zeta potential cell (Zen1010).

#### 2.8.4 Fluorescence Measurements

##### 2.8.4.1 Critical Micelle Concentration (CMC)

The critical micelle concentration values were measured by fluorescence spectroscopy using pyrene as a hydrophobic probe.^17,20,21^ Ten microliters of pyrene stock solution in acetone (0.15 mM) were added to vials, and acetone was removed using an argon flow. Then, 3 mL of aqueous polymer solutions of varying concentrations (10^–4^ to 1 mg·mL^–1^) was introduced to vials. The resulting solutions were left overnight in the dark to reach equilibrium. Then, emission spectra were recorded on a Hitachi F-7000 fluorescence spectrophotometer in the range from 360 to 550 nm (λ_ex_ = 339 nm, the slit width = 2.5 nm). The intensity ratios of the emission peaks at 373 and 394 nm (*I*_373_/*I*_394_) were plotted as a function of the polymer concentration. The CMC values were deducted from the eight data points intersecting the linear regression line of the linearly dependent region.

##### 2.8.4.2 Determination of the Quantity of Encapsulated Doxorubicin in Polymeric Nanoparticles

The amount of encapsulated doxorubicin was determined according to a previously described method.^21,22^ A calibration curve was measured with different concentrations (10^–5^ to 10^–2^ mg·mL^–1^) of DOX solutions in PBS. Emission spectra were recorded within a range of 460–700 nm with an excitation wavelength of 490 nm and a slit width of 5 nm. Doxorubicin-loaded polymeric nanoparticles were dissolved in PBS (1 mg·mL^–1^), and fluorescence measurements were performed. Unfortunately, the small amounts of encapsulated DOX made determining the drug release profile impossible.

##### 2.8.4.3 Fluorescence Spectra of Dye-Labeled Polymers

Fluorescence spectra were measured in PBS at a concentration of 0.1 mg·mL^–1^ (λ_ex_ = 494 nm, λ_em_ = 518 nm, slit width = 2.5 nm).

#### 2.8.5 Size Exclusion Chromatography (SEC)

Size exclusion chromatography (SEC) was employed to determine polymers’ average molar masses and molar mass distributions. THF was used as an eluent (1.0 mL·min^–1^ at 25 °C). Polymers were dissolved in the eluent (5 mg·mL^–1^) and filtered through a 0.45 μm PTFE filter. The samples were analyzed using a two-column set Styragel HR3 and HR4 (Waters) coupled with a three-detector system: refractometer thermostated at 35 °C (Optilab Rex, Wyatt technology), a UV detector (Prostar, Varian) set at 254 nm, and a multiangle laser light scattering detector (Mini Dawn, 3 angles, Wyatt technology). The d*n*/d*c* of the copolymers were calculated based on the weight fraction of PNIPAM (0.107)^23^ or PNVCL (0.137)^24^ and diglyceride-based block. The d*n*/d*c* of PGlyP (0.076 mL·g^–1^) was measured at 620 nm by using a DNDC-2010 differential refractometer.

#### 2.8.6 Mass Spectrometry (MS-ESI)

Spectra were recorded with an Agilent 6530 Accurate-Mass Q-TOF ESI.

#### 2.8.7 Medium-Pressure Liquid Chromatography (MPLC)

Purification with MPLC was performed using a Buchi MSDF 2000 system equipped with a C-605 pump and C-660 fraction collector.

#### 2.8.8 Nuclear Magnetic Resonance (NMR)

The ^1^H and ^13^C NMR spectra were obtained with a Bruker Avance II 400 spectrometer operating at 400 MHz, using CDCl_3_ solutions with TMS as the internal standard.

#### 2.8.9 Transmission Electron Microscopy (TEM)

TEM imaging was performed according to a previously described procedure^15^ using a Tecnai G2 X-Twin microscope.

#### 2.8.10 Thermogravimetric Analyses (TGA)

Samples (2–3 mg) placed in aluminum oxide crucibles were heated in a Mettler Toledo Star TGA/DSC apparatus from 50 to 600 °C (heating rate −10 °C·min^–1^, an argon flow rate −40 mL· min^–1^) using an empty pan as a reference.

#### 2.8.11 Turbidimetry

The measurements were performed according to a previously described procedure^15^ using a Jasco V-670 Spectrophotometer with the Peltier system.

### 2.9 Biological Evaluation

#### 2.9.1 Cytotoxicity Studies

##### 2.9.1.1 Cell Culture

In vitro experiments were performed on the commercial human cell lines THP-1, MCF-7, MDA-MB-231, and H9C2(2-1) according to the protocol provided by ATCC. In brief, monocytic THP-1 cells were grown in RPMI-1640 cell culture medium (ATCC, USA) containing 10% fetal bovine serum (Eurx, Poland), 1% penicillin–streptomycin (Gibco, Life Technologies, Germany) and 0.05 mM 2-mercaptoethanol (Gibco, Life Technologies, Germany). To cultivate breast cancer MCF-7 and MDA-MB-231 cells as well as H9C2(2-1) cardiomyocytes, Dulbecco Modified Eagle Medium (DMEM) containing 10% FBS, and 1% penicillin/streptomycin was used. Cells were grown under controlled temperature conditions at 37 °C and atmosphere at 5% CO_2_.

##### 2.9.1.2 Cell Viability

The cultured cells were exposed to polymeric carriers for 24 h, and then, the effect of tested agents on the proliferative potential was assessed. The viability of THP-1 cells was evaluated by the MTS assay according to the manufacturer’s instructions. In brief, 10 μL of MTS solution was added to cultured cell samples to obtain the final concentration of 0.5 mg·mL^–1^ and incubated for 3 h to form formazan dyes. The formazan dyes were dissolved in 100 μL of dimethyl sulfoxide (DMSO), and the absorbance of colored samples was estimated spectrophotometrically at 570 nm. The obtained values were normalized for absorbance of control nontreated cells, which were taken as 100%. The viability of breast cancer cells and cardiomyocytes was evaluated by using the Neutral Red test for adherent cells. Initially, cells were exposed to the empty or DOX-loaded carriers at the concentrations of 0.05, 0.1, and 0.5 mg·mL^–1^ for 24 h at 37 °C. Next, cells were incubated with 10 μL of neutral red solution for 2 h and finally washed with neutral red assay fixative. The obtained dyes in tested samples were dissolved in 100 μL of solubilization solution, and the absorbance was estimated at a wavelength of 540 nm. The results were normalized for absorbance of control nontreated cells.

#### 2.9.2 Hemocompatibility Assessment

The hemolysis assay was used to study the hemocompatibility of the tested polymeric carriers. First, red body cells collected from healthy donors and previously confirmed for hematocrit value 5% were exposed to polymeric carriers at the concentrations of 0.05, 0.1, and 0.5 mg·mL^–1^ for 24 h at 37 °C. Next, cells were centrifuged, and the concentration of hemoglobulin in obtained supernatants was assessed spectrophotometrically at 540 nm. The hemolytic capacity of tested agents was compared to the effects of PBS (0% hemolysis) and Triton X-100 (hemolysis 100%). All blood samples were anonymous and collected under the Institutional Review Board of the Medical University of Bialystok approval (R-I-002/254/2019) and informed consent of all subjects.

#### 2.9.3 Interaction of Polymeric Carriers with MCF-7 and MDA-MB-231 Cells

Both MCF-7 and MDA-MB-231 cells were seeded in 24-well plates at a density of 250 × 103 cells per well. Cells were cultured to obtain a confluency level of 80% and then exposed to tested agents at a concentration of 0.01 mg·mL^–1^ for 24 h. Due to the presence of the fluorophore molecule fluorescein isothiocyanate in the structure of the synthesized polymeric carriers, a qualitative and quantitative analysis of the tested samples was carried out by their microscopic visualization and fluorescence measurement.

##### 2.9.3.1 Microscopic Evaluation

After a 24 h incubation of the cells with the fluorescently labeled polymeric carriers, samples were analyzed using an inverted Leica DM IL microscope with LED illumination. The cells were washed 3 times with sterile PBS and fixed with 4% paraformaldehyde for 10 min at 4 °C. The samples were then rinsed 3 times with PBS solution and subjected to microscopic analysis. The analysis of yellow–green fluorescence in the cell culture was the basis for assessing the extracellular and intracellular distribution of evaluated polymeric carriers.

##### 2.9.3.2 Fluorescence Intensity Measurement

The adherent MCF-7 and MDA-MB-231 cells were exposed to fluorescein-conjugated polymeric carriers for 24 h at 37 C and evaluated for fluorescence intensity. The culture medium was removed, and cells were carefully washed 3 times with PBS. Then, samples were analyzed using a Varioskan LUX plate reader (Thermo Scientific) with double excitation and emission monochromator at the wavelength λ_ex_ = 490 and λ_em_ = 520 nm.

##### 2.9.3.3 Mode of Action: Apoptosis/Necrosis Assay

The engagement of apoptotic and necrotic pathways in polymeric carrier-induced cell death was assessed by fluorimetric and bioluminescence analysis of annexin V expression. The tested cells were exposed to a free or encapsulated form of DOX at a concentration of 0.5 mg·mL^–1^ for 24 h at 37 °C. In addition, cells were administered with DOX at a concentration of 4 × 1 μM and used as a positive control. Finally, an equal volume of the detection reagent (100 μL) was used in each sample, and the luminescence and fluorescence signals at the following wavelength of λ_ex_ = 485 and λ_em_ = 530 nm were detected.

#### 2.9.4 Statistical Analysis

Statistical analyses were performed using Statistica 13.3 software (StatSoft Inc., Tulsa, OK, USA). The data were analyzed using standard statistical analyses, including the Student’s *t*-test (for independent samples). The *p*-values lower than 0.05 were considered significant.

## 3 Results and Discussion

### 3.1 Synthesis and Characterization of Monomers with the Diacylglycerol Moiety

**Scheme 1:**
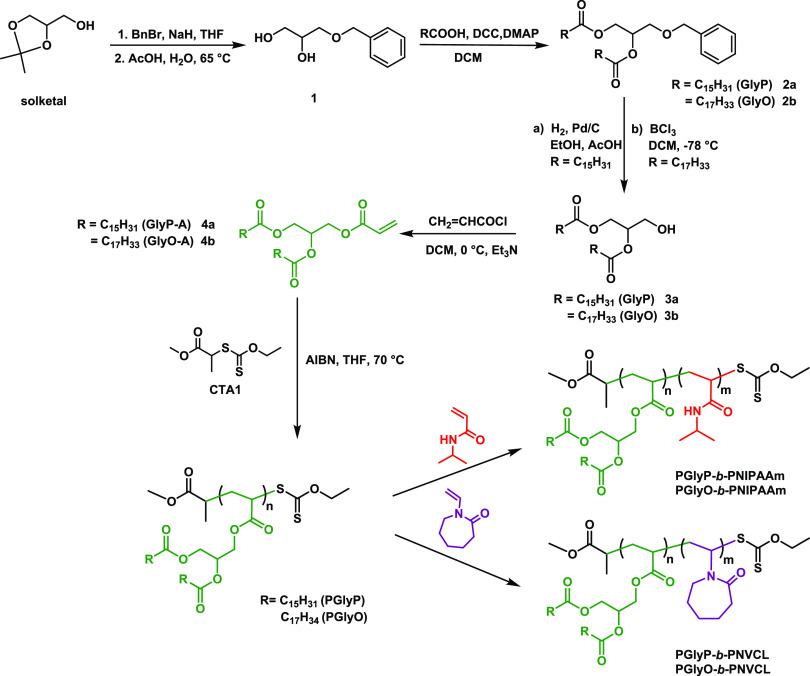
Representation of the Stepwise Synthesis of DAG-Containing Monomers and LPCs by RAFT Polymerization

The DAG-containing monomers were prepared in four synthetic steps (Scheme 1). In the initial step, the −OH group of solketal was protected with benzyl bromide in THF. Next, dioxolane was transformed into corresponding diol **1** by hydrolysis using acetic acid at 65 °C. Subsequently, the synthesis of esters **2a** and **2b**, containing either palmitic or oleic acid, was carried out by Steglich esterification with DMAP as a catalyst and DCC as a coupling agent.^25^ In the case of the derivative with the palmitic acid moiety, the benzyl group was deprotected by hydrogenation to afford the corresponding alcohol (**3a**). In turn, due to the unsaturated bonds in the oleic acid derivative, the hydroxyl function (**3b**) was obtained using BCl_3_ in DCM at −78 °C.^26,27^ Importantly, no side reactions of the oleate double bonds were observed, confirming the effectiveness of the synthetic procedure. The last-step reaction with acryloyl chloride at 0 °C led to the formation of the original diglyceride-based monomers (**4a, 4b**). The monomer containing palmitic acid (**4a**, **GlyP-A**) was obtained as a white solid. In turn, the oleic acid derivative (**4b**, **GlyO-A**) was obtained as a yellowish oil. The products were analyzed by nuclear magnetic resonance (^1^H NMR, ^13^C NMR) and Fourier transform infrared spectroscopy (FT-IR) at each step of the synthesis. The final products were further characterized by mass spectrometry (MS).

The structure of the products after each reaction was proved by NMR spectroscopy (Figures S1 and S2). In the ^1^H NMR spectrum of compound **1**, signals of the benzyl group at 7.5–7.2 and 4.5 ppm were observed. Also, the ^13^C NMR spectrum possessed peaks from the benzyl group at 137.6, 128.4, and 127.7 ppm. After esterification, characteristic signals from the diglyceride segment appeared. ^1^H NMR spectra of **2a** revealed the triplet from FA tails end at 0.9 ppm and the signals from −CH_2_– groups at 1.4 1.3, and 2.6 ppm. Also, the ^13^C NMR spectrum showed new peaks attributed to C=O groups at ∼175 ppm, −CH_2_– groups between 20 and 35 ppm, and −CH_3_ groups at 14 ppm. The effective deprotection of the hydroxyl group in compound **3a** was verified by the disappearance of a peak at 7.5–7.2 ppm in the ^1^H NMR spectrum and the signals in the ^13^C NMR spectrum at 137.6, 128.4, and 127.7 ppm. The ^1^H NMR spectrum of the final monomer (**4a**) exhibited characteristic signals of vinyl protons ranging from 6.4 to 5.9 ppm. Moreover, the ^13^C NMR spectrum displayed a peak from the C=O in the acrylate group at 165.5 ppm, and signals at 131.5 and 127.7 ppm correlated to the acrylate double bond. Similar peaks were also observed for the synthetic route for GlyO (Figures S3 and S4). Nevertheless, due to unsaturated bonds in the oleic moieties in compounds **2b**, **3b**, and **4b**, additional signals were observed at 2.1 and 5.3 ppm in the ^1^H NMR spectra. Also, an additional peak at 130 ppm was present in the ^13^C NMR spectra of these compounds.^28,29^

The synthesized compounds were also analyzed by FT-IR spectroscopy (Figures S5 and S6). The spectrum of compound **1** exhibited a peak at 3350 cm^–1^, indicating the presence of free hydroxyl groups. After the esterification with a fatty acid, the spectrum of compound **2a** displayed new signals, including the bands in the range 2915–2850 and 1450 cm^–1^, associated with C–H stretching and deforming vibrations, respectively. Additionally, distinct bands at 1740 and 1720 cm^–1^ were identified, characteristic of the carbonyl stretching in ester bonds. Acidic hydrolysis was confirmed by the presence of a band at 3350 cm^–1^ in the spectrum of **3a**, which was ascribed to the free hydroxyl groups. The FT-IR spectrum of monomer **4a** showed new peak characteristics of the terminal C=C double bonds at 1635 cm^–1^.^30^ As expected, the FT-IR spectra of derivatives containing palmitic or oleic acid moieties were similar. The only significant difference was the additional band at 3004 cm^–1^ in the spectra of **2b**, **3b**, and **4b** (Figure S6) related to unsaturated bonds.^15,28^

### 3.2 Synthesis and Characterization of Thermoresponsive Polymers with Diglyceride-Based Monomers

**Figure 1:**
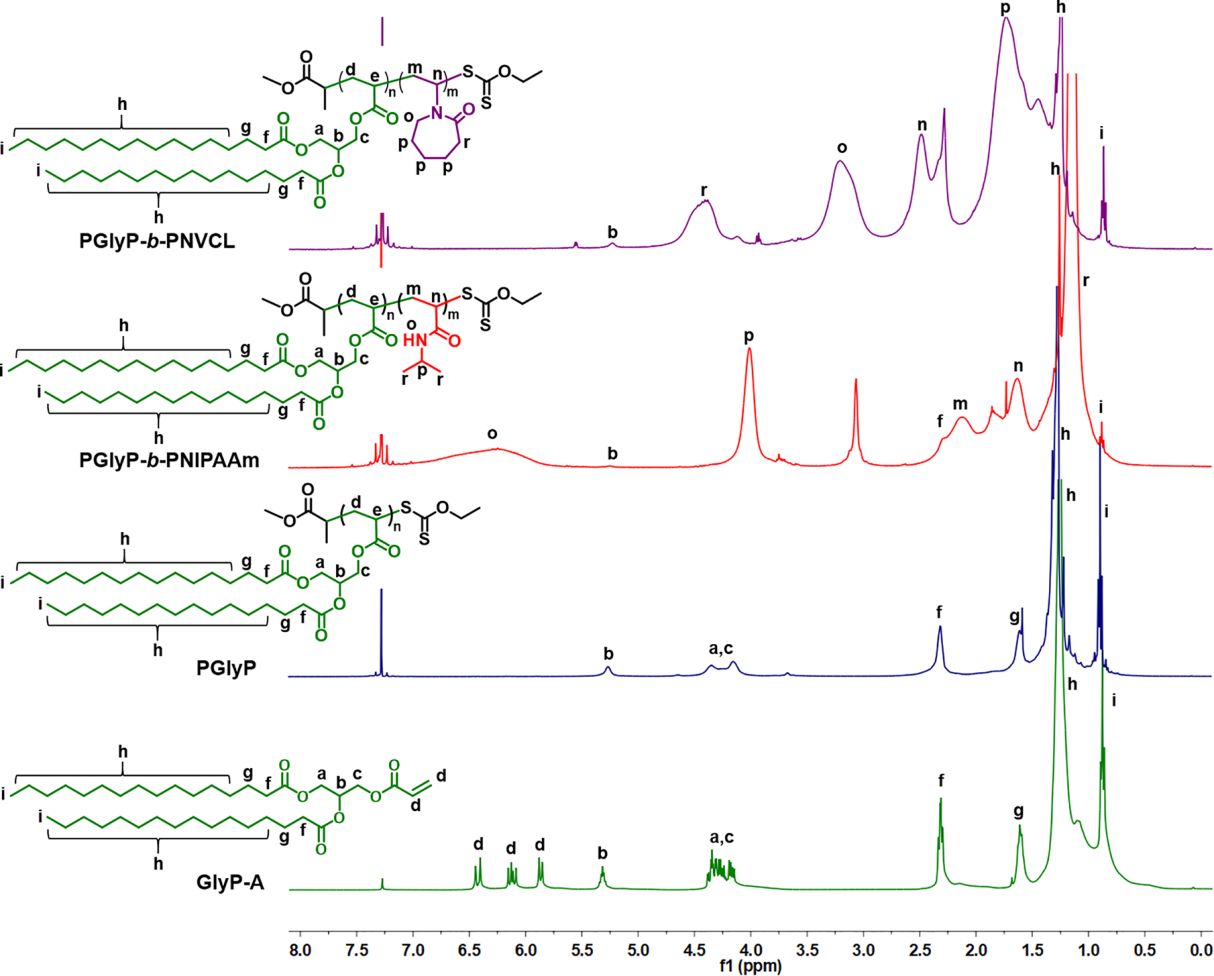
^1^H NMR spectra (400 MHz, CDCl_3_, 298 K) for polymers containing the palmitic acid derivative (GlyP-A).

As shown in Scheme 1, a new class of thermoresponsive lipid-polymer conjugates was synthesized by the RAFT method in two steps. At first, homopolymers of PGlyP (or PGlyO) were obtained using diglyceride-based monomers and well-known methyl 2-((ethoxycarbono-thioyl)thio)propanoate) (CTA1) as a chain transfer agent. Based on the NMR spectra, the monomer conversion for GlyP-A was 93% after 6 h. In the case of PGlyO, polymerization proceeded for 16 h, and the monomer conversion reached 95%. Polymers were further purified by precipitation in cold methanol. NMR and IR spectra indicated successful homopolymerization. The ^1^H NMR spectra showed the disappearance of vinyl proton signals ranging from 5.2 to 5.8 ppm, while peaks broader than those of the corresponding monomers are present (Figures 1 and S7a). As expected, in the case of the FT-IR spectra of the homopolymers, the band ascribed to the acrylate double bond at 1635 cm^–1^ disappeared (Figures S7b and S8).

The homopolymers were characterized by size exclusion chromatography (SEC). Molar masses determined by SEC were in good agreement with the theoretical values (Table 1). Polymers were also characterized by narrow molecular mass distributions (*Đ* < 1.3).

**Table 1:** Synthetic Details and Results of Polymerizations

polymer	[CTA]_0_(mol·L^–1^)	[monomer]_0_(mol·L^–1^)	[AIBN]_0_(mol·L^–1^)	conversion^a^ (%)	*M*_n Th_^b^ (g·mol^–1^)	SEC^c^	
						*M*_n_(g·mol^–1^)	*Đ*
PGlyP	0.0803	0.803	0.530	95	6140	4830	1.17
PGlyO	0.0741	0.741	0.608	93	6480	6320	1.26
PGlyP-*b*-PNIPAAm	0.0167	4.425	0.00167	93	32650	33960	1.40
PGlyP-*b*-PNVCL	0.0167	3.592	0.00668	93	32700	19820	1.33
PGlyO-*b*-PNIPAAm	0.0167	4.425	0.00167	88	32670	28360	1.31
PGlyO-*b*-PNVCL	0.0167	3.59	0.00668	89	32990	19020	1.35

a Calculated from ^1^H NMR in CDCl_3_.

b *M*_n_,_Th_ = [Monomer]_0_/[CTA]_0_·*M*_Monomer_·Conv. + *M*_CTA_*.*

c Measured by SEC-RI-MALS. All polymerizations were conducted with AIBN as an initiator in THF at 70 °C.

In the second step, NIPAAm or NVCL was polymerized in the presence of PGlyP or PGlyO as macro-CTAs. The experimental data for these reactions are depicted in Table 1. The conversions were >88% in all cases, and copolymers were purified by precipitation in cold diethyl ether or pentane. ^1^H NMR results showed broad signals of the PNIPAAm or PNVCL block. Furthermore, the ^1^H NMR spectra confirmed successful block copolymerization. In the spectra of all copolymers, signals from the diglyceride-based block were observed, such as the triplet from the methyl group at the FA chain-end at 0.9 ppm and the peak from the −CH_2_– groups at 1.35 ppm (Figures 1 and S7A). FT-IR analysis (Figure S7A, **8**) of the copolymers showed a trend similar to that of NMR results. The FT-IR spectra of copolymers containing PNIPAAm exhibited broad bands at 3285, 1635, and 1530 cm^–1^, correlated to the stretching of N–H and C=O (amide I) and bending of N–H (amide II) bonds, respectively. For copolymers based on PNVCL, a characteristic carbonyl band in the lactam at 1630 cm^–1^ was noted, along with the signals from the stretching vibrations of the C–N at 1480 cm^–1^ and CH_2_ at 1440 cm^–1^.^15,31^ In FT-IR spectra of all copolymers, the band at ∼1740 cm^–1^ confirmed the presence of a diglyceride-based block.

**Figure 2:**
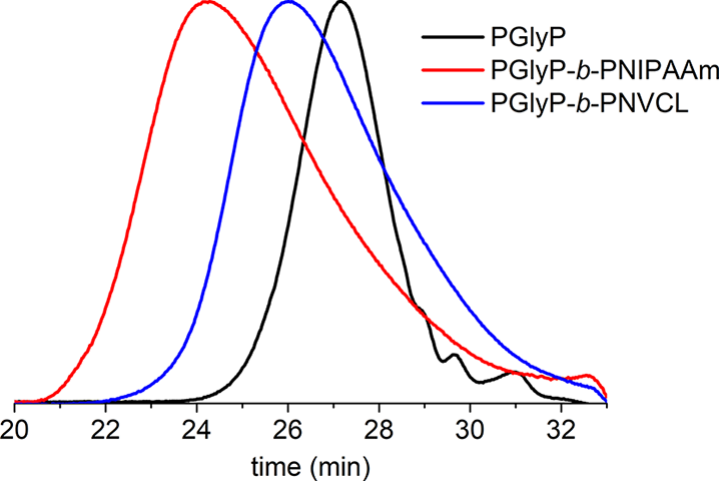
SEC-RI chromatograms of polymers containing the palmitic acid derivative.

The copolymers were characterized by SEC. For all the copolymers, molar mass distributions were narrow (*Đ* < 1.4). For PNIPAAm-based copolymers, good agreement was observed between the molar mass obtained from SEC and the theoretical *M*_n_. However, in the cases of PNVCL-based copolymers, the *M*_n_ values obtained from SEC were lower than the theoretical ones (Table 1). This discrepancy can be explained by chain transfer to the solvent, which sets an upper limit on the attainable molar mass. However, for all samples, a shift to lower elution time was observed in the SEC-RI chromatograms of the block copolymers compared to that of the PGlyP and PGlyO substrates. This observation indicated the complete transformation of the diglyceride-based macroCTAs into block copolymers (Figures 2 and S9).

The thermogravimetric analysis of the PGlyP and PGlyO polymers showed their one-step decomposition in the temperature range of 350–450 °C. The highest decomposition rate was at 400 °C for PGlyP and 405 °C for PGlyO. For the copolymers, one slight weight loss (<5%) up to 200 °C and one significant weight loss (>90%) in the range of 350–470 °C were observed. The former is related to removing the absorbed moisture and the latter to depolymerization. The maximal decomposition rate differed depending on the type of copolymer. It was 415 °C for PNIPAAm-containing copolymers and 435 °C for those with the PNVCL block (Figure S10 and Table S1). The values of glass transition temperature (*T*_g_) determined by DSC for four synthesized copolymers are close to the ones known for PNIPAAm or PNVCL^15^ (Table S1). The *T*_g_ values of PGlyP and PGlyO could not be determined by the applied method. However, DSC measurements revealed differences in the thermal and structural features of both homopolymers. In the case of PGlyP, the peaks related to the melting and crystallization of the sample were observed on the DSC heating and cooling curves, respectively (Figure S11).

Copolymers synthesized in this work consist of hydrophilic (PNIPAAm or PNVCL) and hydrophobic segments (diglyceride of palmitic or oleic acid), allowing for their self-organization in an aqueous solution. The hydrophilic moiety facilitates hydration and swelling, while the hydrophobic components reduce water interactions due to their unfavorable energetic nature.^32^ The copolymers designed in this work showed a critical micelle concentration (CMC). The CMC values were calculated based on fluorescence measurement from the plot of the intensity ratio of pyrene versus polymer concentration. The observed CMC value was ∼7 × 10^–3^ mg·mL^–1^ for the copolymers containing PNVCL (PGlyP-*b*-PNVCL and PGlyO-*b*-PNVCL) and ∼1.4 × 10^–2^ mg·mL^–1^ for those based on PNIPAAm (PGlyP-*b*-PNIPAAm, PGlyO-*b*-PNIPAAm) (Table 2 and Figure S12). The lower CMC values for copolymers containing PNVCL block can be related to the higher hydrophilicity of PNIPAAm compared to PNVCL.^15,33,34^ Nevertheless, the type of lipidated hydrophobic block did not significantly impact the CMC values. Considering that the designed polymer nanoparticles will be further used for biomedical applications and may be diluted, e.g., in body fluids), all experiments on polymeric particles were carried out well above the designated CMCs.

**Table 2:** Summary of Physicochemical Parameters of the Polymers and Polymeric Nanoparticles

polymer	CMC(mg·mL^–1^)	size by number^a^ (nm)	zeta potential^a^ (mV)	*T*_CP_^b^ (°C)	*T*_agg_^c^ (°C)
PGlyP-*b*-PNIPAAm_NP	0.014	30.94 ± 1.95	–6	33.0 ± 0.5	30.5 ± 0.5
PGlyP-*b*-PNIPAAm_NPDOX		41.56 ± 1.38	–5	33.5 ± 0.5	30.5 ± 0.5
PGlyP-*b*-PNVCL_NP	0.007	28.21 ± 1.89	–3	33.0 ± 0.5	31.0 ± 0.5
PGlyP-*b*-PNVCL_NPDOX		44.06 ± 3.38	–5	34.0 ± 0.5	31.5 ± 0.5
PGlyO-*b*-PNIPAAm_NP	0.018	30.94 ± 0.96	–7	33.5 ± 0.5	30.5 ± 0.5
PGlyO-*b*-PNIPAAm_NPDOX		37.47 ± 1.80	–5	33.5 ± 0.5	30.5 ± 0.5
PGlyO-*b*-PNVCL_NP	0.007	23.15 ± 3.73	–3	34.0 ± 0.5	31.5 ± 0.5
PGlyO-*b*-PNVCL_NPDOX		28.70 ± 1.11	–7	34.0 ± 0.5	32.5 ± 0.5
PNIPAAm				34.5 ± 0.5	31 ± 0.5
PNVCL				44.5 ± 0.5	41 ± 0.5

a Measured after 7 days in PBS (0.5 mg·mL^–1^).

b Cloud point temperature measured by turbidimetry.

c Aggregation temperature measured by DLS.

### 3.3 Polymeric Nanoparticle Formation and Characterization

Polymeric nanoparticles were prepared via a simple nanoprecipitation method. Briefly, polymers were dissolved in THF and added dropwise to deionized water with continuous stirring. Next, the resulting solutions were dialyzed against water to eliminate the organic solvent followed by freeze-drying. The obtained nanoparticles were analyzed by multiangle dynamic light scattering (MADLS). To evaluate the colloidal stability of the polymeric nanoparticles, MADLS measurements were taken in water and PBS after 24 h, 7 days, and 30 days. The particle size distributions are shown in Tables 2 and S1.

**Figure 3:**
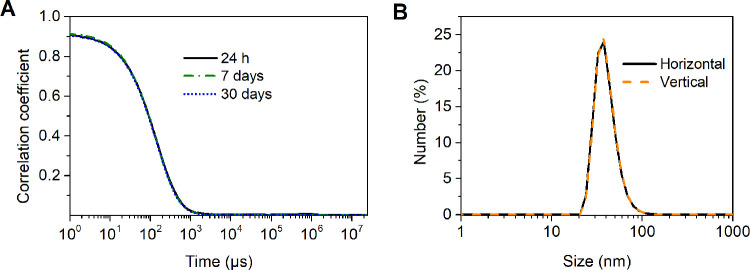
Characteristics of polymeric nanoparticles. (A) Correlograms of PGlyP-*b*-PNIPAAm_NP obtained from DLS experiments with a scattering angle of 173° (PBS, 25 °C, *C* = 1 mg·mL^–1^). (B) Horizontal and vertical measurement for PGlyP-*b*-PNIPAAm_NP (PBS, 25 °C, *C* = 1 mg·mL^–1^).

Regardless of the solvent type, the colloidal stability of all solutions was excellent, and the correlograms showed high similarity even after one month of storage (Figure 3A and Table S2). Finally, the shape of the obtained particles was determined by using dynamic light scattering (DLS) with horizontally and vertically polarized light. The plots acquired from both measurements were superposed, which indicates the formation of spherical nanoparticles (Figure 3B). As shown in Figure S13, TEM imaging confirmed the morphology of the polymeric structures. The monodisperse, spherical shapes were observed.

The ability to form polymeric nanoparticles combined with the long-term stability of the systems at pH 7.4 indicates that they are promising candidates for further biomedical applications. With these results, we further explored the potential of the designed systems as drug carriers. For this purpose, doxorubicin, an anticancer drug with a low water solubility, was selected as a model molecule.

### 3.4 Doxorubicin Encapsulation and Characterization of Drug-Loaded Nanoparticles

The incorporation of doxorubicin into polymeric nanoparticles was conducted via the nanoprecipitation method. During this process, the hydrophobic segment of the copolymer forms an inner core capable of encapsulating drugs with a low water solubility. The quantity of encapsulated doxorubicin in polymeric nanoparticles was determined by the spectrofluorometric method based on the standard curve equation.^21,22^ The values of encapsulated DOX were 0.095, 0.156, 0.148, and 0.247 μg·mL^–1^ for PGlyP-*b*-PNIPAAm_NPDOX, PGlyP-*b*-PNVCL_NPDOX, PGlyO-*b*-PNIPAAm_NPDOX, and PGlyO-*b*-PNVCL_NPDOX, respectively.

The obtained drug-loaded nanoparticles were further analyzed by MADLS. The size of the drug-loaded nanoparticles was slightly larger than that of their blank counterparts (Table 2). This can be explained by increasing the inner space of polymeric nanoparticles after drug encapsulation.^35,36^ In addition, the shape of the particles after drug encapsulation did not change and remained spherical. The observed zeta potential for empty and drug-loaded polymeric nanoparticles was negative in the range of −3 to −7 mV. Regarding surface charge, it is reported that neutral and slightly negative particles (defined as having zeta potentials of −10 to +10 mV) have the longest half-lives in circulation.^37−39^

### 3.5 Thermoresponsive Properties

PNIPAAm and PNVCL are thermoresponsive polymers showing a lower critical solution temperature (LCST) in water. In the following research step, we determined aggregation temperatures (*T*_agg_) by DLS and the cloud point temperatures (*T*_CP_) by turbidimetry. Analyses were performed in PBS at a concentration of 0.5 mg·mL^–1^. The results are presented in Table 2 and Figure S14. Regardless of the technique used, a sharp separation transition was observed. For all copolymers, the phase separation temperatures were lower than those of PNIPAAm or PNVCL. Compared to diglyceride end-capped PNVCLs and PNIPAAms, incorporating diglycerides as repeating units into the polymer structure did not significantly affect the value of the cloud point temperatures.^15^

### 3.6 Biological Studies

In order to explore the potential of the synthesized block copolymers containing membrane-active segment (based on diacylglycerols of palmitic or oleic acid) and thermoresponsive segment (PNIPAAm or PNVCL), selected healthy cells such as human RBC cells, monocytic/macrophage, and myocardial cells as well as estrogen-dependent and estrogen-independent human breast cancer cells were treated with different doses of the empty and drug-loaded carriers. In effect, the relationship between the chemical structure of the synthesized polymeric carriers and their efficacy at the in vitro level was assessed.

**Figure 4:**
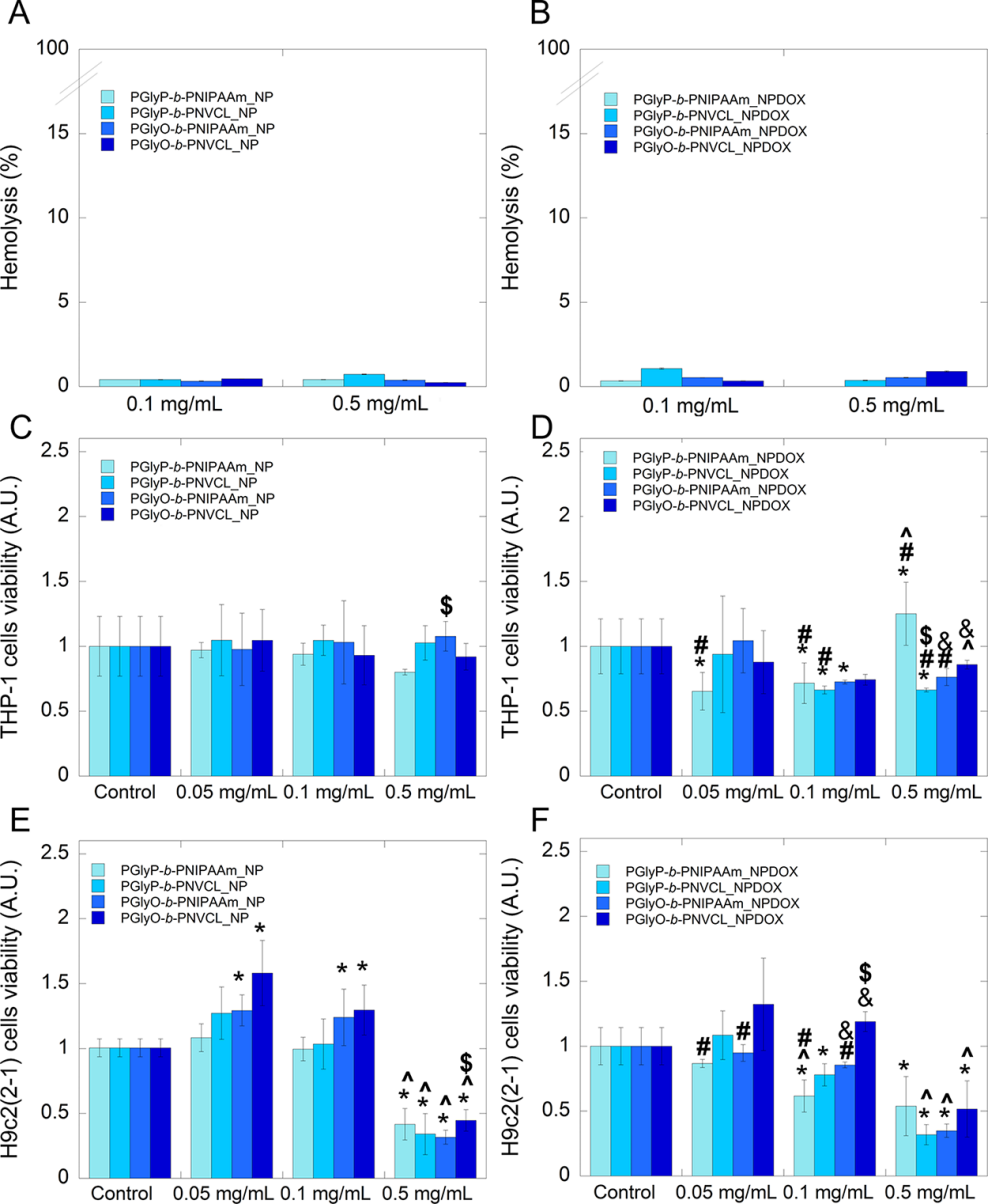
Hemolytic activity and compatibility study of block copolymers containing diacylglycerol-based segment. Hemolytic activity of empty (A) and DOX-loaded (B) carriers containing diacylglycerol-based segment. Viability of monocytic THP-1 cells after addition of empty (C) and DOX-loaded (D) carriers containing diacylglycerol-based segment. Viability of cardiomyocyte H9c2(2-1) cells after the addition of empty (E) and DOX-loaded (F) carriers containing the diacylglycerol-based segment. Statistical significance for the bare carriers or DOX-loaded carriers vs control was marked with (*); the concentration-dependent effect was marked with (^); the comparison of bare carriers vs DOX-loaded carriers was marked with (#). Comparison of PNIPAAm-based carriers vs PNVCL-based carriers was marked with ($); comparison of PGlyP-based carriers vs PGlyO-based carriers was marked with (&), *p* ≤ 0.05. The data presented constitute average results from three measurements ± SD.

At the start of our investigation, the hemocompatibility of the carriers, empty and DOX-loaded, was determined (Figure 4). Results indicated that the incubation of carriers independently of the presence of the chemotherapeutic agent did not cause the loss of the structural integrity of RBCs membranes (Figure 4A,B). The evaluation has shown that the percentage of released hemoglobin was around 1%, classifying proposed drug carriers as nonhemolytic materials that also fit the pharmaceutical criteria regarding hemolytic properties.^40,41^ Obtained results are crucial from the clinical point of view since, during anticancer treatment, the incidence of life-threatening hematological consequences such as acute immune hemolytic anemia (AIHA) might occur.^42^

It is established that anticancer treatment provides for the development of monocytopenia, which is associated with the depletion of hematopoietic cells, including the monocytic germ.^43^ Our studies showed that the addition of empty polymeric nanoparticles to THP-1 monocytic cells did not significantly decrease its proliferation. In contrast, the presence of DOX in the tested carriers caused inhibition of THP-1 cell division. Statistical analysis of the results showed that DOX-loaded PNIPAAm-based carriers containing diacylglycerols of palmitic acid caused a significant impact on THP-1 viability if compared with empty carriers as well as to control cells. In the case of DOX-loaded PNVCL-based carrier containing diacylglycerols of palmitic acid, a marked decrease of metabolic activity if compared to control cells and unloaded carriers has been detected in concentrations 0.1 and 0.5 mg·mL^–1^. A similar observation has been noted after the treatment of the monocytic cells by a DOX-loaded PNIPAAm-based carrier containing diacylglycerols of oleic acid. The disparate effect was indicated in the case of a DOX-loaded PNVCL-based carrier with an oleic moiety. A lack of marked inhibition of cell proliferation compared to that of untreated control cells has been observed. Irrelevant was also the presence of a chemotherapeutic agent in the structure of carriers. However, performing the interpretation based on the ISO 10993-5 norm, cytotoxicity might be classified as weak if the percentage of viable cells is maintained between 80 and 60% after treatment by a tested agent.

Doxorubicin therapy is associated with cardiotoxicity, which might be manifested as occult changes in the myocardial structure and function or as cardiomyopathy and congestive heart failure, requiring cardiac transplantation or causing patient death. In this study, the viability of cardiomyocyte cells after treatment by empty and DOX-loaded carriers has been examined. Results indicated that bare carriers with palmitic acid moieties at concentrations 0.05 and 0.1 mg·mL^–1^ did not significantly influence the viability compared to untreated control. On the other hand, the addition of polymers containing diacylglycerols of oleic acid at these concentrations (0.05 and 0.1 mg·mL^–1^) caused a statistically marked increase in the percentage of survived cells. The 5 times higher concentration of the carriers caused a significant reduction of the percentage of viable cells in a dose depending on the manner for all tested carrier structures. However, compared with other tested carriers, the statistically highest viability has been noted for carriers containing PNVCL and diacylglycerols of oleic acid. The encapsulation of DOX into the structures of the synthesized carriers changed their biological activity. The presence of a chemotherapeutic agent caused a significant depletion in the viability of cells treated with PNIPAAm-containing carriers at concentrations 0.05 and 0.1 mg·mL^–1^. It is worth mentioning that at a concentration of 0.1 mg·mL^–1,^ statistically higher viability has been noted for polymers bearing oleic acid moieties than those containing palmitic acid ones. In turn, if thermoresponsive segments were compared, PNVCL showed higher compatibility with cardiomyocyte cells. Increasing concentration up to 0.5 mg·mL^–1^ caused a dose-dependent reduction of the percentage of viable myocardial cells. However, this effect has not been detected in the case of carriers containing PNIPAAm and diacylglycerols of palmitic acid. It suggests that this type of carrier will not be attractive for biological purposes due to its low compatibility.

A further goal of this research was to study the potential of the prepared carriers as a drug delivery system dedicated to anthracycline antibiotics. Searching for new compounds that exhibit anticancer properties or might modulate or enhance the activity of known antineoplastic agents is a great challenge and an emerging need in modern medicine.^44,45^ The abovementioned is especially important in the context of the growing number of cases of cancer as well as increasing multidrug-resistance phenomena. Furthermore, the reduction of nonspecific interaction of chemotherapeutic agents, which usually causes depletion of the quality of patients’ lives, can be considered a good characteristic for the development of new approaches to anticancer treatment.^46^

**Figure 5:**
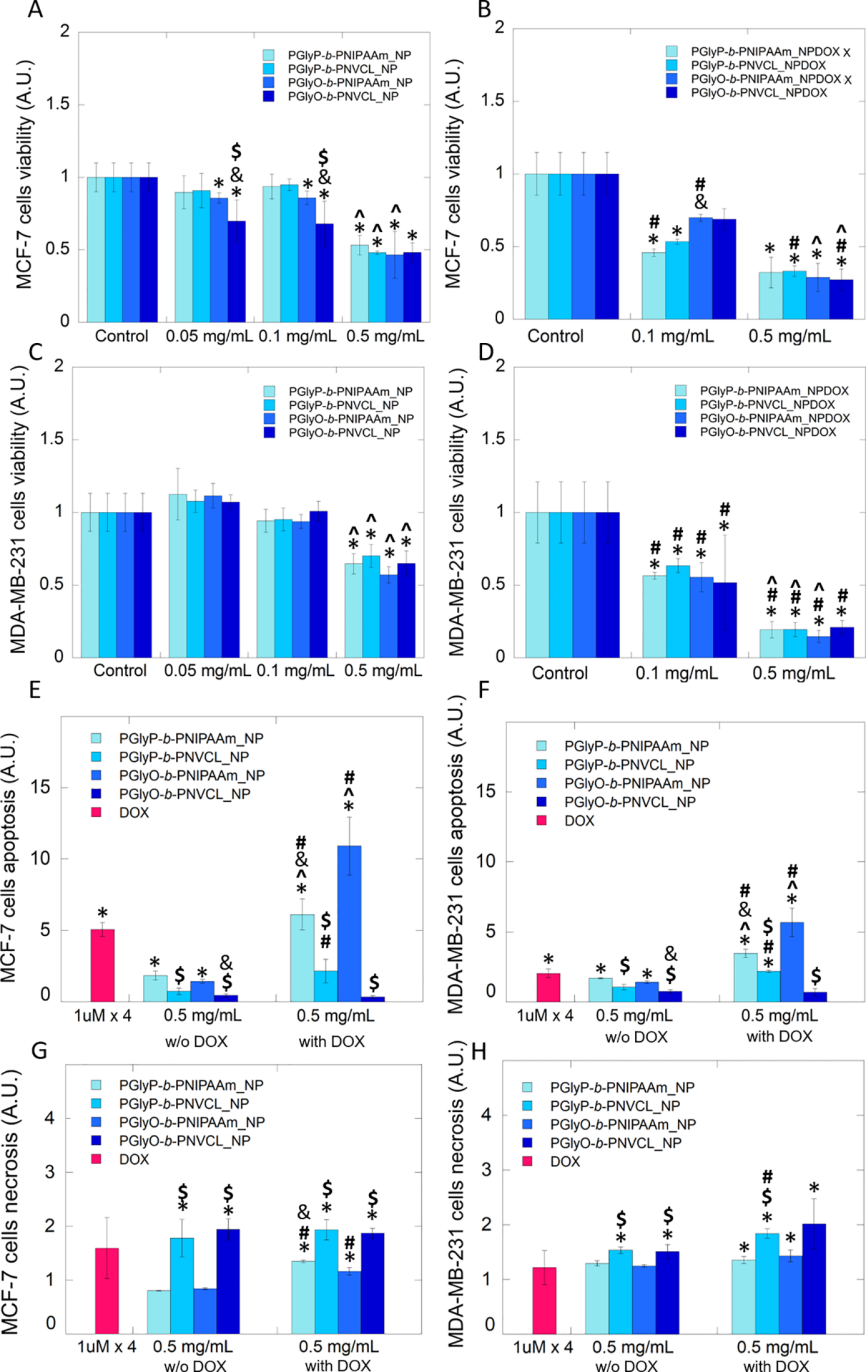
Toxic effect and apoptosis/necrosis induction of the DOX containing polymeric nanoparticles against estrogen-dependent and estrogen-independent breast cancer cells. Viability of estrogen-dependent (A, B) and estrogen-independent (C, D) breast cancer cells in the presence of the empty and DOX-loaded carriers, respectively. Induction of apoptosis or necrosis in MCF-7 (E, G) and MDA-MB-231 (F, H) cells after the addition of DOX, carriers without DOX (w/o DOX), and DOX-loaded carriers (with DOX). Statistical significance for the bare carriers or DOX-loaded carriers vs control was marked with (*); the concentration-dependent effect for apoptosis and necrosis evaluation vs DOX at free form was marked with (^); was marked with (^); comparison of bare carriers vs DOX-loaded carriers was marked with (#). Comparison of PNIPAAm-based carriers vs PNVCL-based carriers was marked with ($); comparison of PGlyP-based carriers vs PGlyO-based carriers was marked with (&) *p* ≤ 0.05. The data presented constitute average results from three measurements ± SD.

The anticancer activity of prepared polymeric nanoparticles as drug carriers for DOX delivery against two different breast cancer cell lines was investigated. It is established that the MCF-7 cell line is noninvasive, and its growth depends on estrogen and epidermal growth factor (EGF). In contrast, MDA-MB-231 cells are known as a model for more aggressive, hormone-independent breast cancer with terrible prognosis for the patients.^47^ These two breast cancer cell lines were treated by tested carriers (empty and DOX-loaded) applied at the concentrations of 0.05, 0.1, and 0.5 mg·mL^–1^ and incubated for 24 h. As shown in Figure 5A,C, carriers comprising the PGlyP block did not exert a cytotoxic effect against both examined cell lines. The percentage of viable cells, around 90%, was noted if carriers were applied at concentrations of 0.05 and 0.1 mg·mL^–1^. In contrast, PGlyO-containing carriers caused a statistically significant depletion of the viable MCF-7 cells. Moreover, a marked reduction of survived cells was indicated if cells were treated by carriers composed of PGlyO and PNVCL blocks compared to treatment by PGlyO-*b*-PNIPAAm. It can be concluded that at concentrations of 0.05 and 0.1 mg·mL^–1^, in the case of MCF-7 cells, a significant difference in cytotoxic activity has depended not only on the presence of unsaturated acid moieties but also on the thermoresponsive segment. The 5-fold increase in the carriers’ concentration indicated a significant decrease in viability in a dose-dependent manner compared to control for both evaluated cell lines.

**Figure 6:**
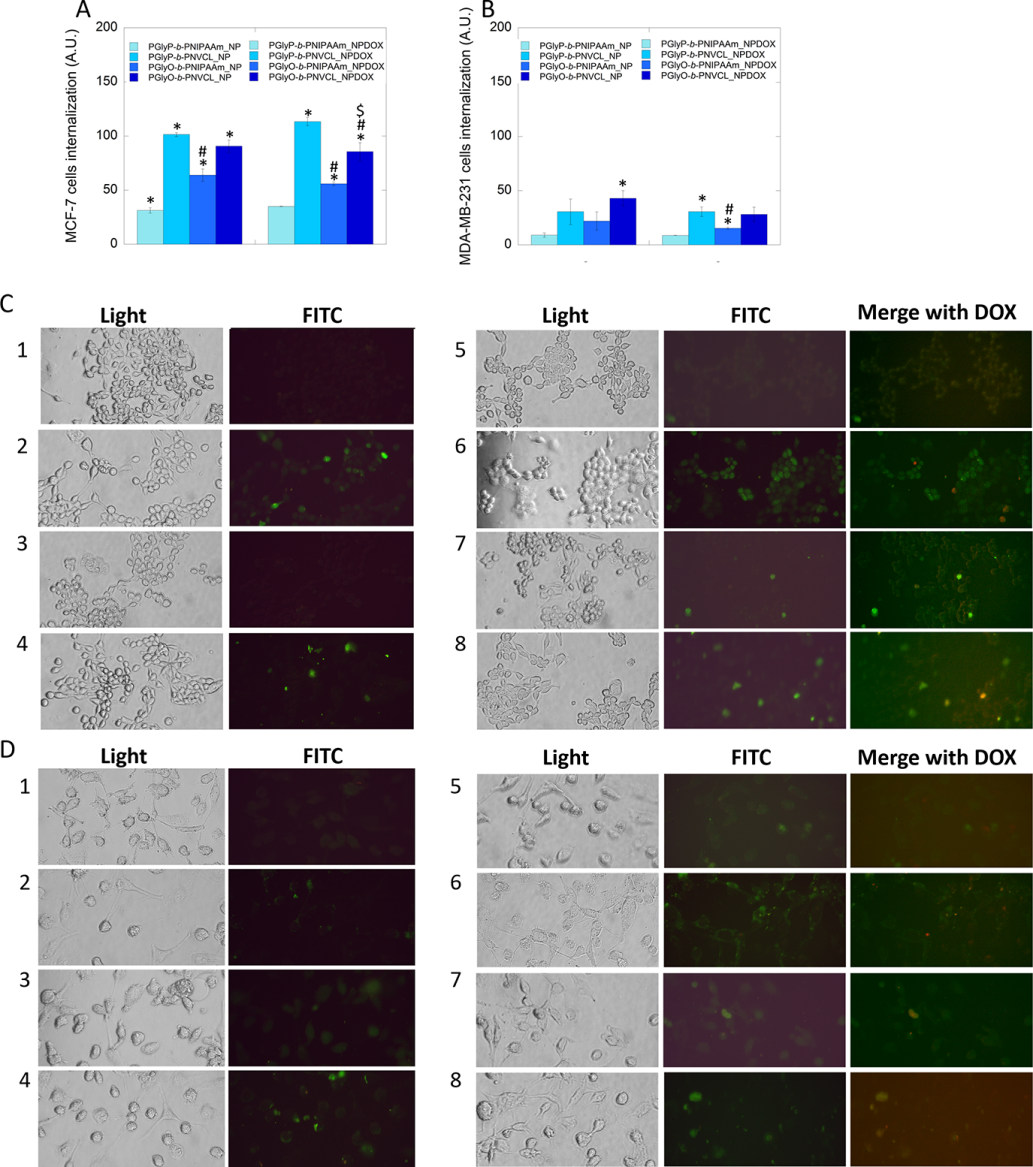
Internalization of fluorescein-labeled empty and DOX-loaded carriers into breast cancer cells. Fluorescence intensity of fluorescein-labeled empty and DOX-loaded carriers in MCF-7 (A) and MDA-MB-231 (B) cells. Microscopic analysis of fluorescein-labeled empty and DOX-loaded carriers localization into MCF-7 (C) and MDA-MB-231 (D) cells. 200× magnification. Statistical significance for the bare carriers or DOX-loaded carriers vs PGlyP-*b*-PNIPAAm marked by (*); vs PGlyP-*b*-PNVCL marked by (#); vs PGlyO-*b*-PNIPAAm marked by ($), *p* ≤ 0,05. Numbers: (1) PGlyP-*b*-PNIPAAm_NP; (2) PGlyP-*b*-PNVCL_NP; (3) PGlyO-*b*-PNIPAAm_NP; (4) PGlyO-*b*-PNVCL_NP; (5) PGlyP-*b*-PNIPAAm_NPDOX; (6) PGlyP-*b*-PNVCL_NPDOX; (7) PGlyO-*b*-PNVCL_NPDOX; (8) PGlyP-*b*-PNIPAAm _NPDOX.

The only exception was the PGlyO-*b*-PNVCL-based carrier, in the case of which the lack of dose-dependent efficacy has been detected. This suggests that a lower carrier concentration might be used, while the efficacy will be similar to the one noted for a higher dose. In effect, using a lower dose of carriers will provide higher compatibility and decrease the risk of developing the side effects of therapy. Results shown in Figure 5B,D indicate that the formulation of carriers containing DOX caused a strong cytotoxic effect. After the addition of the DOX-loaded carriers at concentrations 0.1 and 0.5, a statistically significant depletion of cell viability by 40–55% and by 70–85%, respectively, in comparison to the untreated control, has been observed. Depending on the presence of DOX in the carriers, in the case of estrogen-depend cells, a statistically significant reduction of the percentage of viable cells has been noted for carriers containing PNIPAAm if applied at a concentration of 0.1 mg·mL^–1^ and for PNVCL-based carriers if applied at concentration 0.5 mg·mL^–1^. Additionally, for the carriers bearing diacylglycerols of oleic acid, the dose-dependent effect was detected if they were used at higher concentrations. In turn, in the case of representatives of the aggressive breast cancer cell line—MDA-MB-231, treatment by DOX-loaded carriers exerts significantly better efficacy for all tested carriers than that of empty polymers. The observed effect is statistically dependent on applied concentration, excluding PGlyO-*b*-PNVCL_NPDOX carriers. Moreover, in all cases, a lack of structure–activity relationship has been detected in estrogen-independent breast cancer cells. Bioluminescent-based assays were performed to evaluate whether the treated cells die via the apoptosis pathway. During the analysis, Annexin V luciferase fusion proteins can bind to phosphatidylserine (PS), which is exposed to the outer leaflet of the cell during early apoptosis. Results presented in Figure 5E,F indicate that, after 24 h exposition in both kinds of treated cells, bare and DOX-loaded PNIPAAm-based carriers caused statistically significant apoptosis induction if compared to untreated cells and cells treated by PNVCL-based carriers. If the impact of the diacylglycerol moiety on apoptosis induction is compared, in the case of bare carriers, a higher level of apoptotic cells was noted after treatment by carriers with the palmitic moiety. However, in the case of loaded carriers, the effect depended on the thermoresponsive part. In brief, for PNIPAAm-based carriers, a 2-fold higher percentage of apoptotic cells was observed for systems containing oleic moieties. In the case of PNVCL-based carriers, a higher efficacy in apoptosis induction was observed for systems with saturated acid moieties. Noteworthy is that the treatment of MCF-7 cells and MDA-MB-231 cells with DOX-loaded polymeric carriers caused similar or 2-fold increased apoptosis if compared to drugs applied in the free form at a concentration of 4 μM, which is many times higher than the DOX content in the evaluated carriers. In another set of experiments, we investigated whether necrosis-associated cell death is involved in the observed cytotoxicity. For this purpose, a fluorescent-based assay was employed, which measures the signal after the loss of membrane integrity and binds the dyes with DNA. As indicated in Figure 6G,H, the synthesized carriers that contain PNVCL segments, empty and loaded with DOX, can induce necrotic cell death more effectively than carriers with PNIPAAm block and at a similar level as a drug in free form. Interestingly, in the case of estrogen-dependent cells, the presence of DOX in all evaluated carriers increased the ability to induce necrotic-based cell death. This suggests that the carriers’ mechanism of action depends on the presence of PNVCL or the PNIPAAm block. The abovementioned suggestion might explain the lower ability of PNVCL-based carriers to induce apoptosis in the treated cells. However, while apoptosis and necrosis are the main pathways through which cancer cells die, the question of which way should cancer treatment be targeted remains open to debate. Apoptosis is a natural process in all cells and may help the body’s immune system fight tumor cells. In turn, necrosis can trigger an immune response that potentially inhibits the body’s natural immune defenses to fight cancer.^45^

### 3.7 Modification of the Polymer Chain End with a Fluorescent Probe

To understand how polymeric delivery systems act inside biological systems, we modified the polymer chain end with a fluorescent moiety. We applied a two-step postpolymerization labeling procedure. First, the terminal dithiocarbonate groups were reduced to thiol moiety by simple aminolysis with propylamine in the presence of tributylphosphine.^48^ The complete removal of the dithiocarbonate group was confirmed by UV–vis spectroscopy by the disappearance of the characteristic absorption band at 280 nm (Figure S14). Polymers terminated with the −SH group were further modified with a fluorescein diacetate 5-maleimide using Michael addition.^49^

Dye-labeled polymers were then used to prepare lipid–polymer nanoparticles (empty and drug-loaded) by the nanoprecipitation method described earlier. We studied the fluorescence properties of linear fluorescein-labeled polymers and the corresponding polymeric nanoparticles (Figure S15). In all cases, the fluorescence intensity was higher for polymeric nanoparticles, which is related to the accumulation of dye molecules in a smaller space.^50^

The results of carriers’ internalization into estrogen-dependent and estrogen-independent cells are summarized in Figure 6. Microscopic analysis (Figure 6C,D) showed that carriers with the PNVCL block indicated better penetration than carriers built with the PNIPAAm segment. Fluorescence-based studies (Figure 6A,B) are in agreement with the microscopic results. It can be postulated that the thermoresponsive block might determine cellular internalization. Additionally, statistical analysis revealed that in the case of PNIPAAm-based carriers, the diacylglycerol part modulates carrier internalization. It has been observed that the presence of unsaturated acid in PGlyO-*b*-PNIPAAm-based carriers increased their internalization into the cells.

## 4 Conclusions

This study presents the synthesis of original block copolymers containing membrane-active segments (based on diacylglycerols of palmitic or oleic acid) and thermoresponsive segments (PNIPAAm or PNVCL). Using RAFT polymerization, well-defined amphiphilic block copolymers with targeted molecular masses and narrow molecular mass distributions were obtained. Subsequently, these copolymers were formed into polymeric nanoparticles (NPs) with and without doxorubicin. The obtained NPs have a diameter of several tens of nanometers and a slightly negative zeta potential. Their phase transition temperature in water might be tuned by changing the polymer composition. They are nonhemolytic and do not show activity against THP-1 monocytic cells and H9c2(2-1) cardiomyocyte cells. All of the above factors make them good candidates for drug delivery systems. The empty and DOX-loaded NPs show structure-dependent activity toward the selected cells. DOX-loaded NPs were able to internalize into cells and cause a marked reduction in the viability of breast cancer cells, including a highly aggressive and invasive triple-negative MDA-MB-231 cell line. A real-time luminescence and fluorescent assay was performed to study the mode of action of the synthesized carriers. It confirmed their ability to induce apoptosis or necrosis-associated cell death in the treated cells.

## Supporting Information Available

Complementary characterization data for all products (monomers, polymers, polymeric nanoparticles), including NMR, DSC, TG, FT-IR, SEC, TEM, and fluorescence (PDF)

## References

[ref1] Sung Y. K.; Kim S. W. Recent Advances in Polymeric Drug Delivery Systems. Biomater. Res. 2020, 24 (1), 12 10.1186/s40824-020-00190-7. 32537239 PMC7285724

[ref2] Kumar R.; Santa Chalarca C. F.; Bockman M. R.; Bruggen C. V.; Grimme C. J.; Dalal R. J.; Hanson M. G.; Hexum J. K.; Reineke T. M. Polymeric Delivery of Therapeutic Nucleic Acids. Chem. Rev. 2021, 121 (18), 11527–11652. 10.1021/acs.chemrev.0c00997. 33939409

[ref3] Bochicchio S.; Lamberti G.; Barba A. A. Polymer–Lipid Pharmaceutical Nanocarriers: Innovations by New Formulations and Production Technologies. Pharmaceutics 2021, 13 (2), 198 10.3390/pharmaceutics13020198. 33540659 PMC7913085

[ref4] Wannasarit S.; Wang S.; Figueiredo P.; Trujillo C.; Eburnea F.; Simón-Gracia L.; Correia A.; Ding Y.; Teesalu T.; Liu D.; Wiwattanapatapee R.; Santos H. A.; Li W. A Virus-Mimicking PH-Responsive Acetalated Dextran-Based Membrane-Active Polymeric Nanoparticle for Intracellular Delivery of Antitumor Therapeutics. Adv. Funct. Mater. 2019, 29 (51), 1905352 10.1002/adfm.201905352.

[ref5] Alves A. C.; Ribeiro D.; Nunes C.; Reis S. Biophysics in Cancer: The Relevance of Drug-Membrane Interaction Studies. Biochim. Biophys. Acta, Biomembr. 2016, 1858 (9), 2231–2244. 10.1016/j.bbamem.2016.06.025. 27368477

[ref6] Stewart M. P.; Langer R.; Jensen K. F. Intracellular Delivery by Membrane Disruption: Mechanisms, Strategies, and Concepts. Chem. Rev. 2018, 118 (16), 7409–7531. 10.1021/acs.chemrev.7b00678. 30052023 PMC6763210

[ref7] Goñi F. M.; Alonso A. Structure and Functional Properties of Diacylglycerols in Membranes1This Work Is Dedicated to Professor Vittorio Luzzati on Occasion of His 75th Birthday.1. Prog. Lipid Res. 1999, 38 (1), 1–48. 10.1016/S0163-7827(98)00021-6. 10396601

[ref8] Campomanes P.; Zoni V.; Vanni S. Local Accumulation of Diacylglycerol Alters Membrane Properties Nonlinearly Due to Its Transbilayer Activity. Commun. Chem. 2019, 2 (1), 72 10.1038/s42004-019-0175-7.

[ref9] Gómez-Fernández J. C.; Corbalán-García S. Diacylglycerols, Multivalent Membrane Modulators. Chem. Phys. Lipids 2007, 148 (1), 1–25. 10.1016/j.chemphyslip.2007.04.003. 17560968

[ref10] Carrasco S.; Mérida I. Diacylglycerol, When Simplicity Becomes Complex. Trends Biochem. Sci. 2007, 32 (1), 27–36. 10.1016/j.tibs.2006.11.004. 17157506

[ref11] Eichmann T. O.; Lass A. DAG Tales: The Multiple Faces of Diacylglycerol—Stereochemistry, Metabolism, and Signaling. Cell. Mol. Life Sci. 2015, 72 (20), 3931–3952. 10.1007/s00018-015-1982-3. 26153463 PMC4575688

[ref12] Schuhmacher M.; Grasskamp A. T.; Barahtjan P.; Wagner N.; Lombardot B.; Schuhmacher J. S.; Sala P.; Lohmann A.; Henry I.; Shevchenko A.; Coskun Ü.; Walter A. M.; Nadler A. Live-Cell Lipid Biochemistry Reveals a Role of Diacylglycerol Side-Chain Composition for Cellular Lipid Dynamics and Protein Affinities. Proc. Natl. Acad. Sci. U. S. A. 2020, 117 (14), 7729–7738. 10.1073/pnas.1912684117. 32213584 PMC7149225

[ref13] Sen N.; Hause G.; Binder W. H. Membrane Anchored Polymers Modulate Amyloid Fibrillation. Macromol. Rapid Commun. 2021, 42 (12), 2100120 10.1002/marc.202100120. 33987913

[ref14] Watanabe A.; Niu J.; Lunn D. J.; Lawrence J.; Knight A. S.; Zhang M.; Hawker C. J. PET-RAFT as a Facile Strategy for Preparing Functional Lipid-Polymer Conjugates. J. Polym. Sci., Part A: Polym. Chem. 2018, 56 (12), 1259–1268. 10.1002/pola.29007.

[ref15] Kurowska I.; Markiewicz K. H.; Niemirowicz-Laskowska K.; Misiak P.; Destarac M.; Wielgat P.; Misztalewska-Turkowicz I.; Siemiaszko G.; Car H.; Wilczewska A. Z. Membrane-Active Diacylglycerol-Terminated Thermoresponsive Polymers: RAFT Synthesis and Biocompatibility Evaluation. Eur. Polym. J. 2022, 169, 111154 10.1016/j.eurpolymj.2022.111154.

[ref16] Dizman B.; Elasri M. O.; Mathias L. J. Synthesis and Characterization of Antibacterial and Temperature Responsive Methacrylamide Polymers. Macromolecules 2006, 39 (17), 5738–5746. 10.1021/ma0607620.

[ref17] Misiak P.; Niemirowicz-Laskowska K.; Markiewicz K. H.; Misztalewska-Turkowicz I.; Wielgat P.; Kurowska I.; Siemiaszko G.; Destarac M.; Car H.; Wilczewska A. Z. Evaluation of Cytotoxic Effect of Cholesterol End-Capped Poly(N-Isopropylacrylamide)s on Selected Normal and Neoplastic Cells. Int. J. Nanomed. 2020, 15, 7263–7278. 10.2147/IJN.S262582. PMC7533236 33061380

[ref18] Siemiaszko G.; Niemirowicz-Laskowska K.; Markiewicz K. H.; Misztalewska-Turkowicz I.; Dudź E.; Milewska S.; Misiak P.; Kurowska I.; Sadowska A.; Car H.; Wilczewska A. Z. Synergistic Effect of Folate-Conjugated Polymers and 5-Fluorouracil in the Treatment of Colon Cancer. Cancer Nanotechnol. 2021, 12 (1), 31 10.1186/s12645-021-00104-9.

[ref19] Milewska S.; Siemiaszko G.; Wilczewska A. Z.; Misztalewska-Turkowicz I.; Markiewicz K. H.; Szymczuk D.; Sawicka D.; Car H.; Lazny R.; Niemirowicz-Laskowska K. Folic-Acid-Conjugated Thermoresponsive Polymeric Particles for Targeted Delivery of 5-Fluorouracil to CRC Cells. Int. J. Mol. Sci. 2023, 24 (2), 1364 10.3390/ijms24021364. 36674883 PMC9861804

[ref20] Wang H.; Li Z.; Lu S.; Li C.; Zhao W.; Zhao Y.; Yu S.; Wang T.; Sun T. Nano Micelles of Cellulose-Graft-Poly (l-Lactic Acid) Anchored with Epithelial Cell Adhesion Antibody for Enhanced Drug Loading and Anti-Tumor Effect. Mater. Today Commun. 2020, 22, 100764 10.1016/j.mtcomm.2019.100764.

[ref21] Misiak P.; Niemirowicz-Laskowska K.; Markiewicz K. H.; Wielgat P.; Kurowska I.; Czarnomysy R.; Misztalewska-Turkowicz I.; Car H.; Bielawski K.; Wilczewska A. Z. Doxorubicin-Loaded Polymeric Nanoparticles Containing Ketoester-Based Block and Cholesterol Moiety as Specific Vehicles to Fight Estrogen-Dependent Breast Cancer. Cancer Nanotechnol. 2023, 14 (1), 23 10.1186/s12645-023-00176-9.

[ref22] Misiak P.; Niemirowicz-Laskowska K.; Misztalewska-Turkowicz I.; Markiewicz K. H.; Wielgat P.; Car H.; Wilczewska A. Z. Doxorubicin Delivery Systems with an Acetylacetone-Based Block in Cholesterol-Terminated Copolymers: Diverse Activity against Estrogen-Dependent and Estrogen-Independent Breast Cancer Cells. Chem. Phys. Lipids 2022, 245, 105194 10.1016/j.chemphyslip.2022.105194. 35288126

[ref23] Zhou S.; Fan S.; Au-yeung S. C. F.; Wu C. Light-Scattering Studies of Poly(N-Isopropylacrylamide) in Tetrahydrofuran and Aqueous Solution. Polymer 1995, 36 (7), 1341–1346. 10.1016/0032-3861(95)95910-S.

[ref24] Siirilä J.; Häkkinen S.; Tenhu H. The Emulsion Polymerization Induced Self-Assembly of a Thermoresponsive Polymer Poly(*N* -Vinylcaprolactam). Polym. Chem. 2019, 10 (6), 766–775. 10.1039/C8PY01421C.

[ref25] Lee J.-D.; Ueno M.; Miyajima Y.; Nakamura H. Synthesis of Boron Cluster Lipids: *Closo* -Dodecaborate as an Alternative Hydrophilic Function of Boronated Liposomes for Neutron Capture Therapy. Org. Lett. 2007, 9 (2), 323–326. 10.1021/ol062840+. 17217295

[ref26] Bouton J.; Van Hecke K.; Van Calenbergh S. Efficient Diastereoselective Synthesis of a New Class of Azanucleosides: 2′-Homoazanucleosides. Tetrahedron 2017, 73 (30), 4307–4316. 10.1016/j.tet.2017.05.083. 32287431 PMC7111761

[ref27] Du W.; Kulkarni S. S.; Gervay-Hague J. Efficient, One-Pot Syntheses of Biologically Active α-Linked Glycolipids. Chem. Commun. 2007, 23, 2336–2338. 10.1039/B702551C. 17844738

[ref28] Vilela C.; Rua R.; Silvestre A. J. D.; Gandini A. Polymers and Copolymers from Fatty Acid-Based Monomers. Ind. Crops Prod. 2010, 32 (2), 97–104. 10.1016/j.indcrop.2010.03.008.

[ref29] Chira N.; Nicolescu A.; Raluca S.; Rosca S. Fatty Acid Composition of Vegetable Oils Determined from C-13-NMR Spectra. Rev. Chim. 2016, 67, 1257–1263.

[ref30] Gan H.; Hutchinson S. A.; Hurren C.; Liu Q.; Wang X.; Long R. L. Effect of Oleic Purity on the Chemical Structure, Thermal and Rheological Properties of Bio-Based Polymers Derived from High Oleic Cottonseed Oil via RAFT Polymerization. Ind. Crops Prod. 2021, 171, 113882 10.1016/j.indcrop.2021.113882.

[ref31] Kozanoǧlu S.; Özdemir T.; Usanmaz A. Polymerization of N-Vinylcaprolactam and Characterization of Poly(N-Vinylcaprolactam). J. Macromol. Sci. Part A 2011, 48 (6), 467–477. 10.1080/10601325.2011.573350.

[ref32] dos Santos S.; Medronho B.; dos Santos T.; Antunes F. E. Amphiphilic Molecules in Drug Delivery Systems. In Drug Delivery Systems: Advanced Technologies Potentially Applicable in Personalised Treatment; Coelho J., Ed.; Advances in Predictive, Preventive and Personalised Medicine; Springer Netherlands: Dordrecht, 2013; vol 4, pp 35–85.

[ref33] Van Gheluwe L.; Chourpa I.; Gaigne C.; Munnier E. Polymer-Based Smart Drug Delivery Systems for Skin Application and Demonstration of Stimuli-Responsiveness. Polymers 2021, 13 (8), 1285 10.3390/polym13081285. 33920816 PMC8071137

[ref34] Kozlovskaya V.; Kharlampieva E. Self-Assemblies of Thermoresponsive Poly(*N* -Vinylcaprolactam) Polymers for Applications in Biomedical Field. ACS Appl. Polym. Mater. 2020, 2 (1), 26–39. 10.1021/acsapm.9b00863.

[ref35] Zhou Y.; Yu J.; Feng X.; Li W.; Wang Y.; Jin H.; Huang H.; Liu Y.; Fan D. Reduction-Responsive Core-Crosslinked Micelles Based on a Glycol Chitosan–Lipoic Acid Conjugate for Triggered Release of Doxorubicin. RSC Adv. 2016, 6 (37), 31391–31400. 10.1039/C6RA05501J.

[ref36] Sarkar P.; Ghosh S.; Saha R.; Sarkar K. RAFT Polymerization Mediated Core–Shell Supramolecular Assembly of PEGMA- *Co* -Stearic Acid Block Co-Polymer for Efficient Anticancer Drug Delivery. RSC Adv. 2021, 11 (28), 16913–16923. 10.1039/D1RA01660A. 35479720 PMC9031514

[ref37] Mitchell M. J.; Billingsley M. M.; Haley R. M.; Wechsler M. E.; Peppas N. A.; Langer R. Engineering Precision Nanoparticles for Drug Delivery. Nat. Rev. Drug Discovery 2021, 20 (2), 101–124. 10.1038/s41573-020-0090-8. 33277608 PMC7717100

[ref38] Blanco E.; Shen H.; Ferrari M. Principles of Nanoparticle Design for Overcoming Biological Barriers to Drug Delivery. Nat. Biotechnol. 2015, 33 (9), 941–951. 10.1038/nbt.3330. 26348965 PMC4978509

[ref39] Dogra P.; Adolphi N. L.; Wang Z.; Lin Y.-S.; Butler K. S.; Durfee P. N.; Croissant J. G.; Noureddine A.; Coker E. N.; Bearer E. L.; Cristini V.; Brinker C. J. Establishing the Effects of Mesoporous Silica Nanoparticle Properties on in Vivo Disposition Using Imaging-Based Pharmacokinetics. Nat. Commun. 2018, 9 (1), 4551 10.1038/s41467-018-06730-z. 30382084 PMC6208419

[ref40] Amin K.; Dannenfelser R.-M. In Vitro Hemolysis: Guidance for the Pharmaceutical Scientist. J. Pharm. Sci. 2006, 95 (6), 1173–1176. 10.1002/jps.20627. 16639718

[ref41] Totea G.; Ionita D.; Demetrescu I.; Mitache M. M. In Vitro Hemocompatibility and Corrosion Behavior of New Zr-Binary Alloys in Whole Human Blood. Cent. Eur. J. Chem. 2014, 12 (7), 796–803. 10.2478/s11532-014-0535-1.

[ref42] Doll D. C.; Weiss R. B. Hemolytic Anemia Associated with Antineoplastic Agents. Cancer Treat. Rep. 1985, 69 (7–8), 777–782. 3160460

[ref43] Kondo M.; Oshita F.; Kato Y.; Yamada K.; Nomura I.; Noda K. Early Monocytopenia after Chemotherapy as a Risk Factor for Neutropenia. Am. J. Clin. Oncol. 1999, 22 (1), 103–105. 10.1097/00000421-199902000-00025. 10025393

[ref44] Bousbaa H. Novel Anticancer Strategies. Pharmaceutics 2021, 13 (2), 275 10.3390/pharmaceutics13020275. 33670469 PMC7922003

[ref45] Bobrin V. A.; Lin Y.; He J.; Qi Y.; Gu W.; Monteiro M. J. Therapeutic Delivery of Polymeric Tadpole Nanostructures with High Selectivity to Triple Negative Breast Cancer Cells. Biomacromolecules 2020, 21 (11), 4457–4468. 10.1021/acs.biomac.0c00302. 32212644

[ref46] Milewska S.; Niemirowicz-Laskowska K.; Siemiaszko G.; Nowicki P.; Wilczewska A. Z.; Car H. Current Trends and Challenges in Pharmacoeconomic Aspects of Nanocarriers as Drug Delivery Systems for Cancer Treatment. Int. J. Nanomed. 2021, 16, 6593–6644. 10.2147/IJN.S323831. PMC8487283 34611400

[ref47] Nohara K.; Wang F.; Spiegel S. Glycosphingolipid Composition of MDA-MB-231 and MCF-7 Human Breast Cancer Cell Lines. Breast Cancer Res. Treat. 1998, 48 (2), 149–157. 10.1023/A:1005986606010. 9596486

[ref48] Glaria A.; Beija M.; Bordes R.; Destarac M.; Marty J.-D. Understanding the Role of ω-End Groups and Molecular Weight in the Interaction of PNIPAM with Gold Surfaces. Chem. Mater. 2013, 25 (9), 1868–1876. 10.1021/cm400480p.

[ref49] Li M.; De P.; Gondi S. R.; Sumerlin B. S. End Group Transformations of RAFT-generated Polymers with Bismaleimides: Functional Telechelics and Modular Block Copolymers. J. Polym. Sci., Part A: Polym. Chem. 2008, 46 (15), 5093–5100. 10.1002/pola.22837.

[ref50] Reisch A.; Klymchenko A. S. Fluorescent Polymer Nanoparticles Based on Dyes: Seeking Brighter Tools for Bioimaging. Small 2016, 12 (15), 1968–1992. 10.1002/smll.201503396. 26901678 PMC5405874

