# Supplementary material for: Membrane-Active Thermoresponsive Block Copolymers Containing a Diacylglycerol-Based Segment: RAFT Synthesis, Doxorubicin Encapsulation, and Evaluation of Cytotoxicity against Breast Cancer Cells
Source: Biomacromolecules. 2023 Oct 16;24(11):4854–68. doi: 10.1021/acs.biomac.3c00580 (PMC10646981; doi:10.1021/acs.biomac.3c00580)
Supplement: Supplementary file 1 — bm3c00580_si_001.pdf [file bm3c00580_si_001.pdf]

SUPPLEMENTARY INFORMATION

Membrane-active thermoresponsive block copolymers containing diacylglycerol-based segment: RAFT synthesis, doxorubicin encapsulation, and evaluation of cytotoxicity against breast cancer cells

Izabela Kurowska,[a,b] Karolina H. Markiewicz,[a*] Katarzyna Niemirowicz-Laskowska,[c] Mathias Destarac,[d] Przemysław Wielgat,[e] Iwona Misztalewska-Turkowicz,[a] Paweł Misiak,[a] Halina Car,[c,e] Agnieszka Z. Wilczewska[a*]

[a] *Faculty of Chemistry, University of Bialystok, Ciolkowskiego 1K, 15-245 Bialystok, Poland*
[b] *Doctoral School of Exact and Natural Sciences, University of Bialystok, Bialystok, Poland*
[c] *Department of Experimental Pharmacology, Medical University of Bialystok, Szpitalna 37, 15-295 Bialystok, Poland*
[d] *Laboratoire IMRCP, CNRS UMR 5623, Paul Sabatier University, 31062 Toulouse Cedex 09, France*
[e] *Department of Clinical Pharmacology, Medical University of Bialystok, Waszyngtona 15A, 15-274 Bialystok, Poland*

Table of Contents

Figure S1. ^{1}H NMR spectra (400 MHz, $CDCl_3$, 298K) for the synthesis route of monomer containing palmitic acid derivative (GlyP-A). 3

Figure S2. ^{13}C NMR spectra (100 MHz, $CDCl_3$, 298K) for the synthesis route of monomer containing palmitic acid derivative (GlyP-A). 3

Figure S3. ^{1}H NMR spectra (400 MHz, $CDCl_3$, 298K) for the synthesis route of monomer containing oleic acid derivative (GlyO-A). 4

Figure S4. ^{13}C NMR spectra (100 MHz, $CDCl_3$, 298K) for the synthesis route of monomer containing oleic acid derivative (GlyO-A). 4

Figure S5. FT-IR spectra for the synthesis route of monomer containing palmitic acid derivative (GlyP-A). 5

Figure S6. FT-IR spectra for the synthesis route of monomer containing oleic acid derivative (GlyO-A). 5

Figure S7. (a) ^{1}H NMR spectra (400 MHz, $CDCl_3$, 298K) and (b) FT-IR spectra for polymers containing oleic acid derivative (GlyO-A). 7

Figure S8. FT-IR spectra for polymers containing palmitic acid derivative (GlyP-A). 7

Figure S9. SEC-RI chromatograms of polymers containing oleic acid derivative. 7

Figure S10. TG (top panels) and DTG curves (bottom panels) of the studied polymers and copolymers. 8

Figure S11. DSC curves (the second heating and cooling run) of the studied polymers and copolymers. 8

Figure S12. Intensity ratios (I_{394}/I_{373}) from pyrene emission spectra plotted with a concentration of polymers. 9

Figure S13. TEM images for a) PGlyP-*b*-PNIPAAm_NP and b) PGlyO-*b*-PNIPAAm_NP. 9

Figure S14. Evolution of transmittance measured by turbidimetry and number size distribution as a function of temperature for polymeric nanoparticles. 10

Figure S15. UV-Vis spectra of PGlyP-*b*-PNIPAAm terminated with xanthate (solid line) and reduced to thiol (dashed line). 11

Figure S16. Fluorescence properties of polymers and corresponding polymeric nanoparticles. 11

Table S1. Thermal properties of the polymers. 12

Table S2. Evaluation of colloidal stability of polymeric nanoparticles. 12

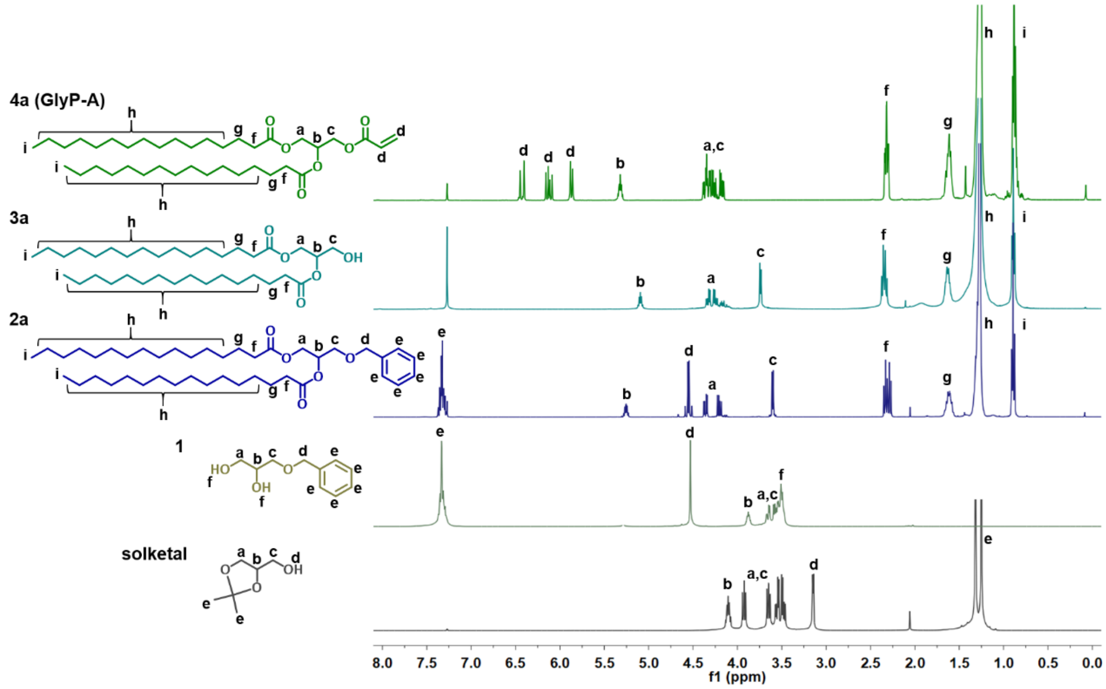

Figure S1. ^{1}H NMR spectra (400 MHz, $CDCl_3$, 298K) for the synthesis route of monomer containing palmitic acid derivative (GlyP-A).

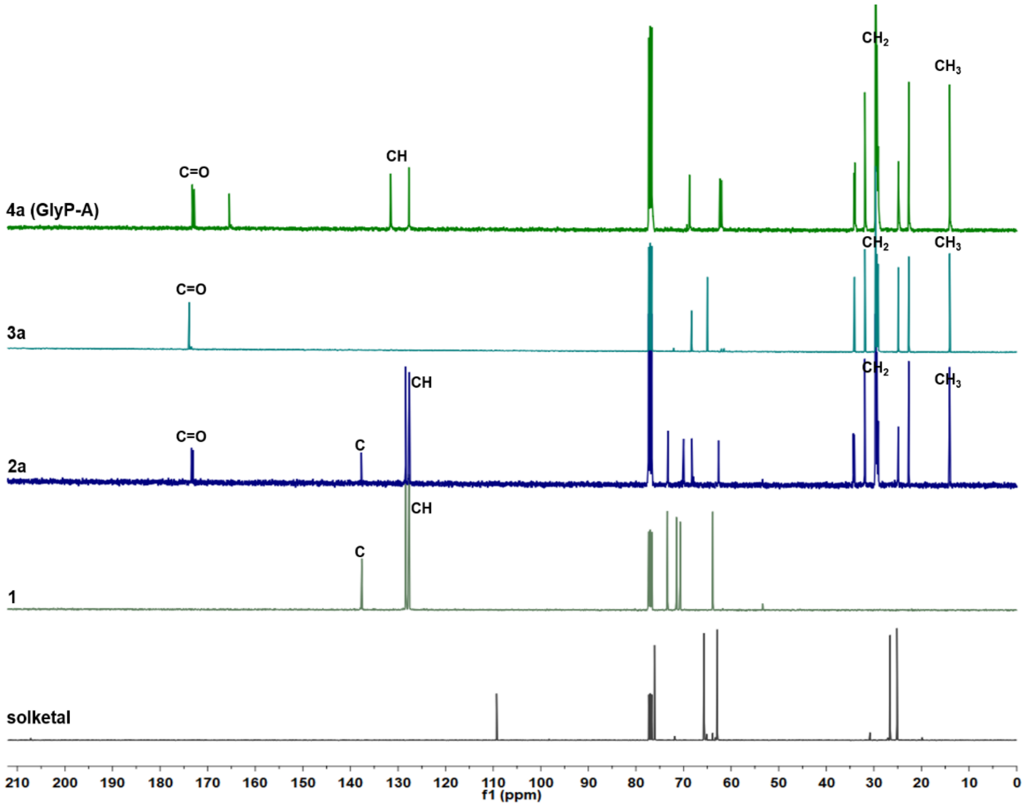

Figure S2. ^{13}C NMR spectra (100 MHz, $CDCl_3$, 298K) for the synthesis route of monomer containing palmitic acid derivative (GlyP-A).

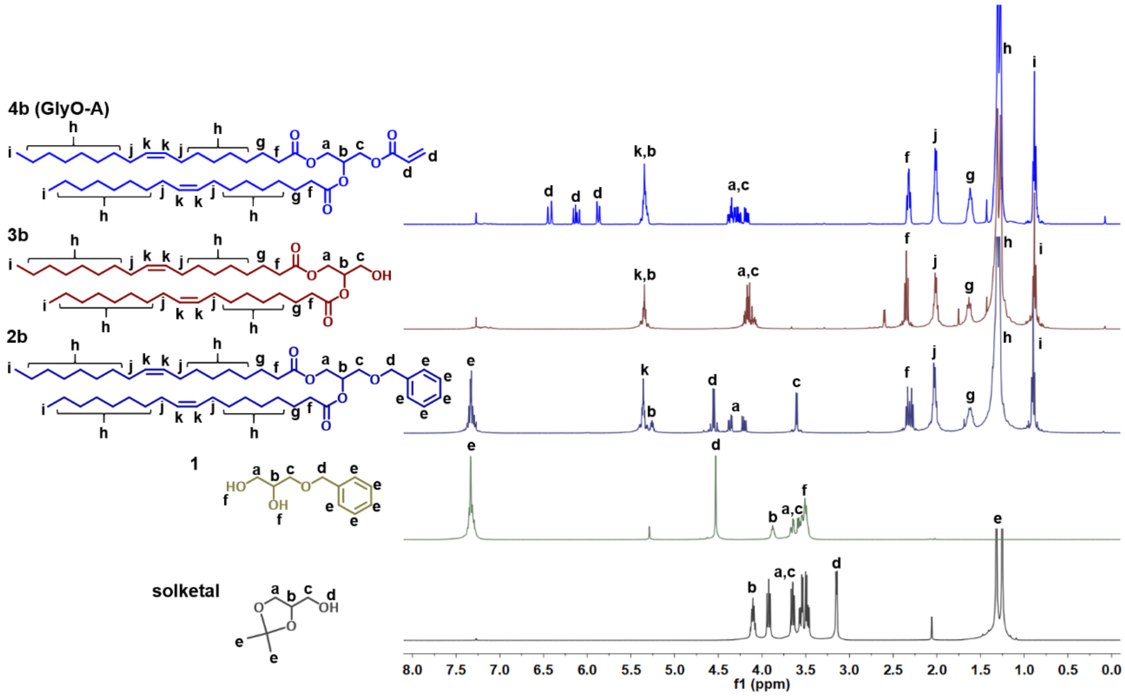

Figure S3. ^{1}H NMR spectra (400 MHz, $CDCl_3$, 298K) for the synthesis route of monomer containing oleic acid derivative (GlyO-A).

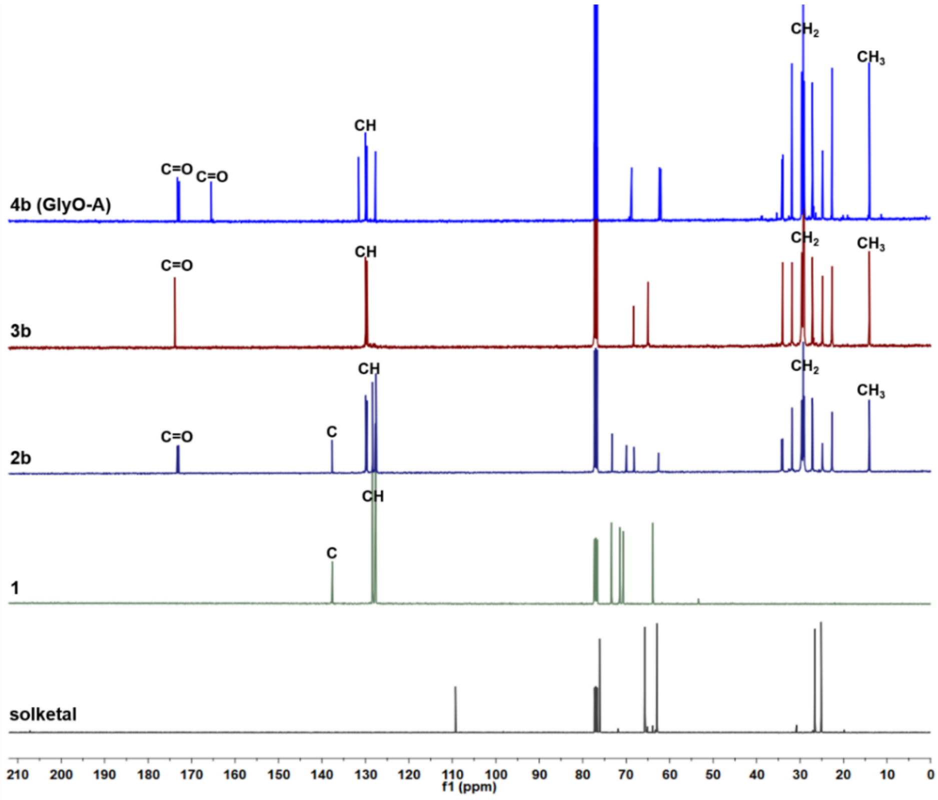

Figure S4. ^{13}C NMR spectra (100 MHz, $CDCl_3$, 298K) for the synthesis route of monomer containing oleic acid derivative (GlyO-A).

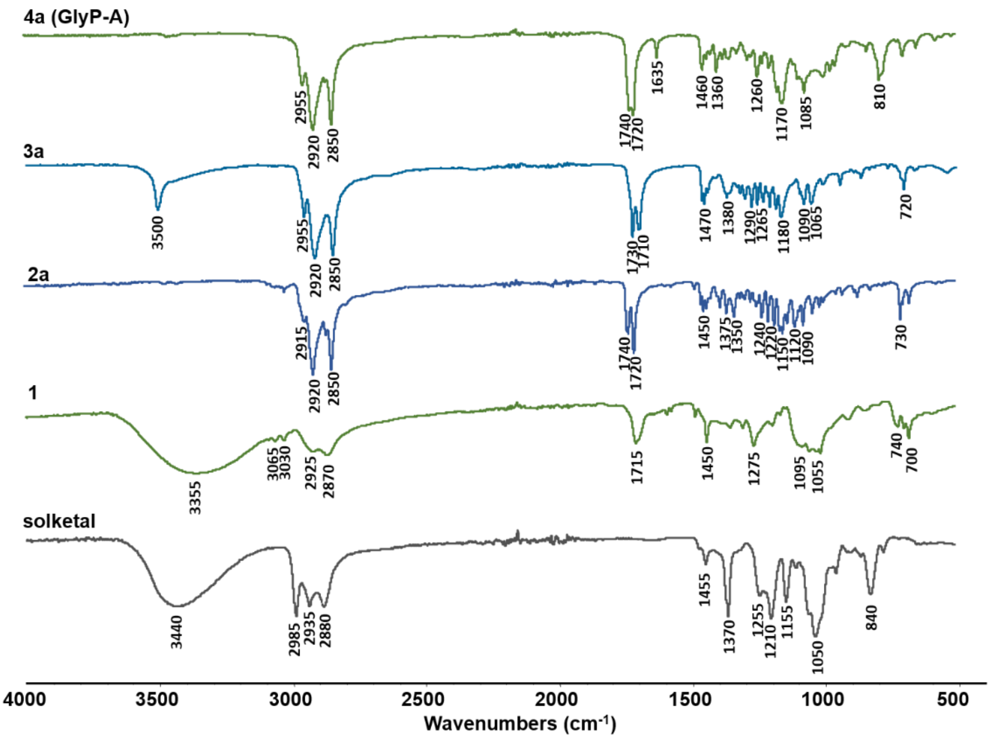

Figure S5. FT-IR spectra for the synthesis route of monomer containing palmitic acid derivative (GlyP-A).

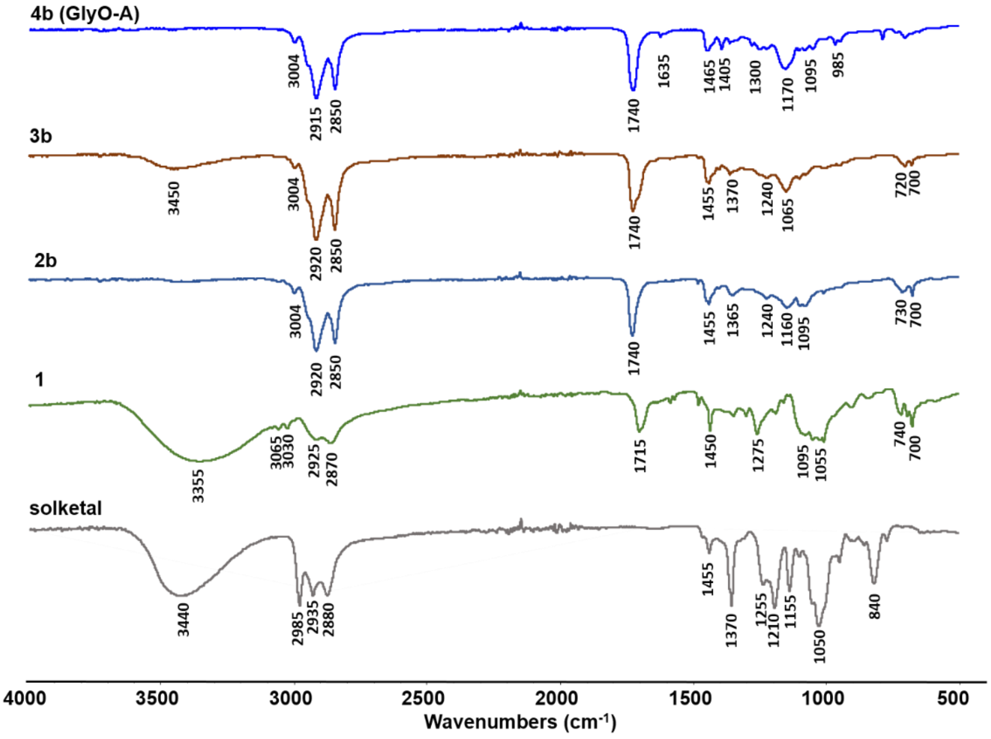

Figure S6. FT-IR spectra for the synthesis route of monomer containing oleic acid derivative (GlyO-A).

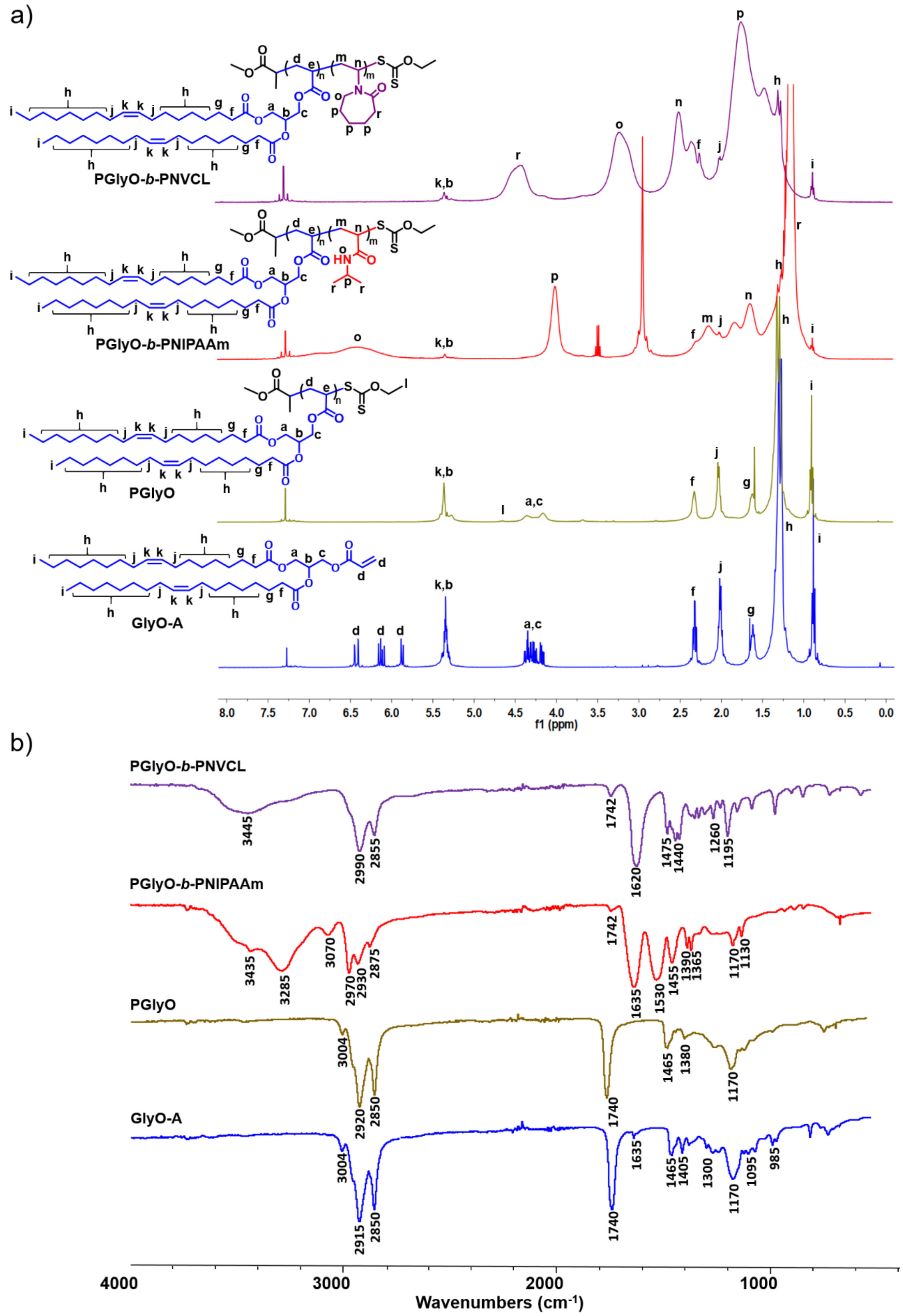
a)
PGlyO-b-PNVCL
PGlyO-b-PNIPAAm
PGlyO
GlyO-A
f1 (ppm)
b)
PGlyO-b-PNVCL
3445
2990
2855
1742
1620
1475
1440
1260
1195
PGlyO-b-PNIPAAm
3435
3285
3070
2970
2930
2875
1742
1635
1530
1455
1390
1365
1170
1130
PGlyO
3004
2920
2850
1740
1465
1380
1170
GlyO-A
3004
2915
2850
1740
1635
1465
1405
1300
1170
1095
985
Wavenumbers (cm-1)

Figure S7. (a) ^{1}H NMR spectra (400 MHz, $CDCl_3$, 298K) and (b) FT-IR spectra for polymers containing oleic acid derivative (GlyO-A).

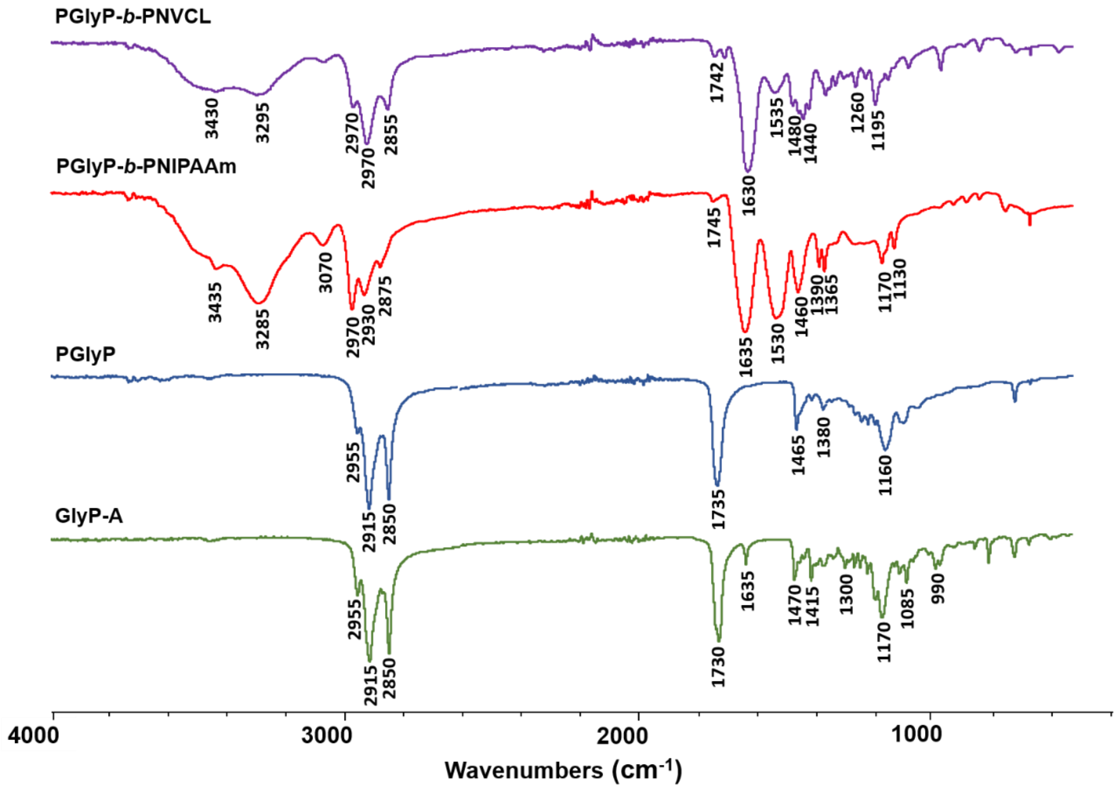

Figure S8. FT-IR spectra for polymers containing palmitic acid derivative (GlyP-A).

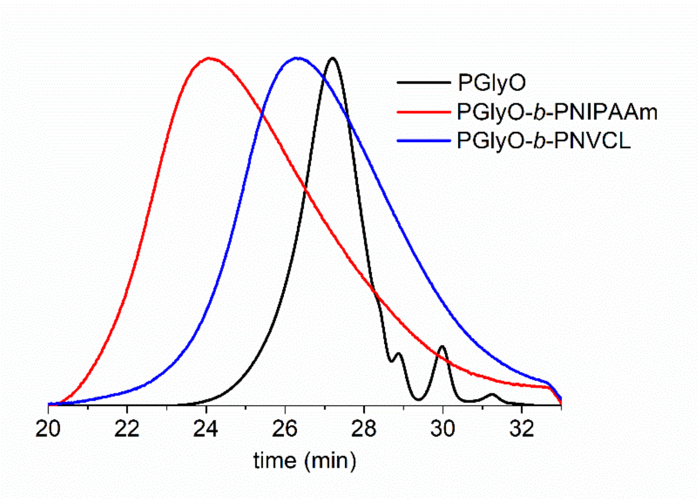

Figure S9. SEC-RI chromatograms of polymers containing oleic acid derivative.

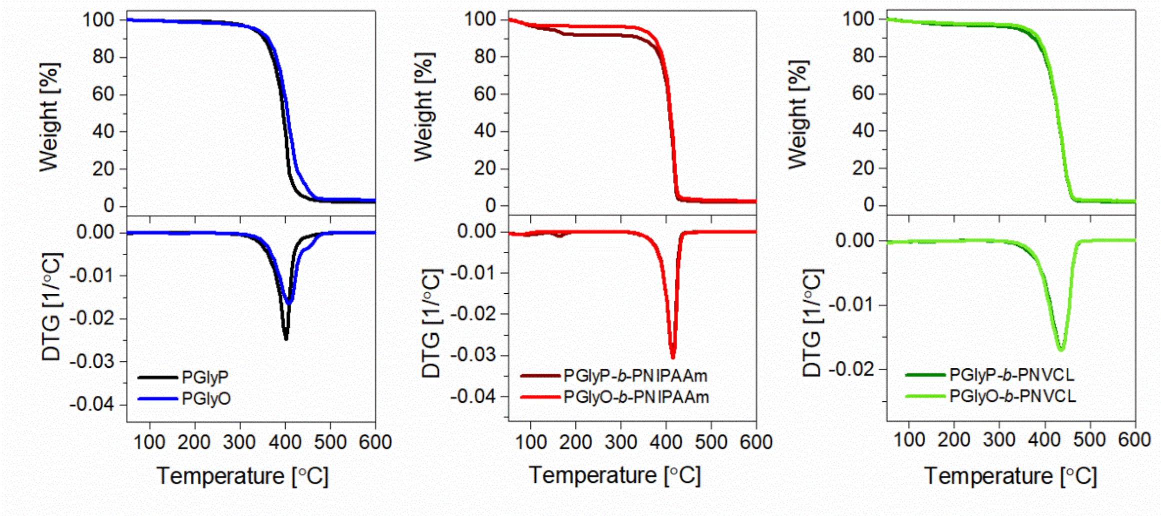

Figure S10. TG (top panels) and DTG curves (bottom panels) of the studied polymers and copolymers.

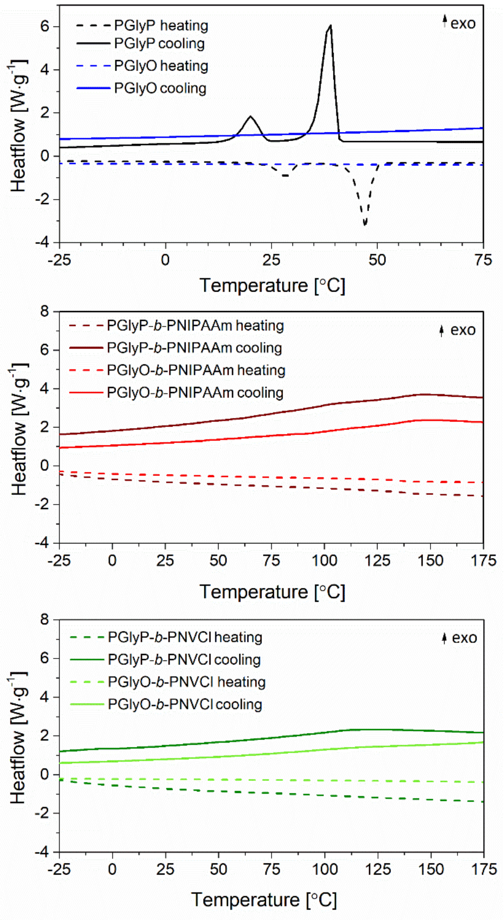

Figure S11. DSC curves (the second heating and cooling run) of the studied polymers and copolymers.

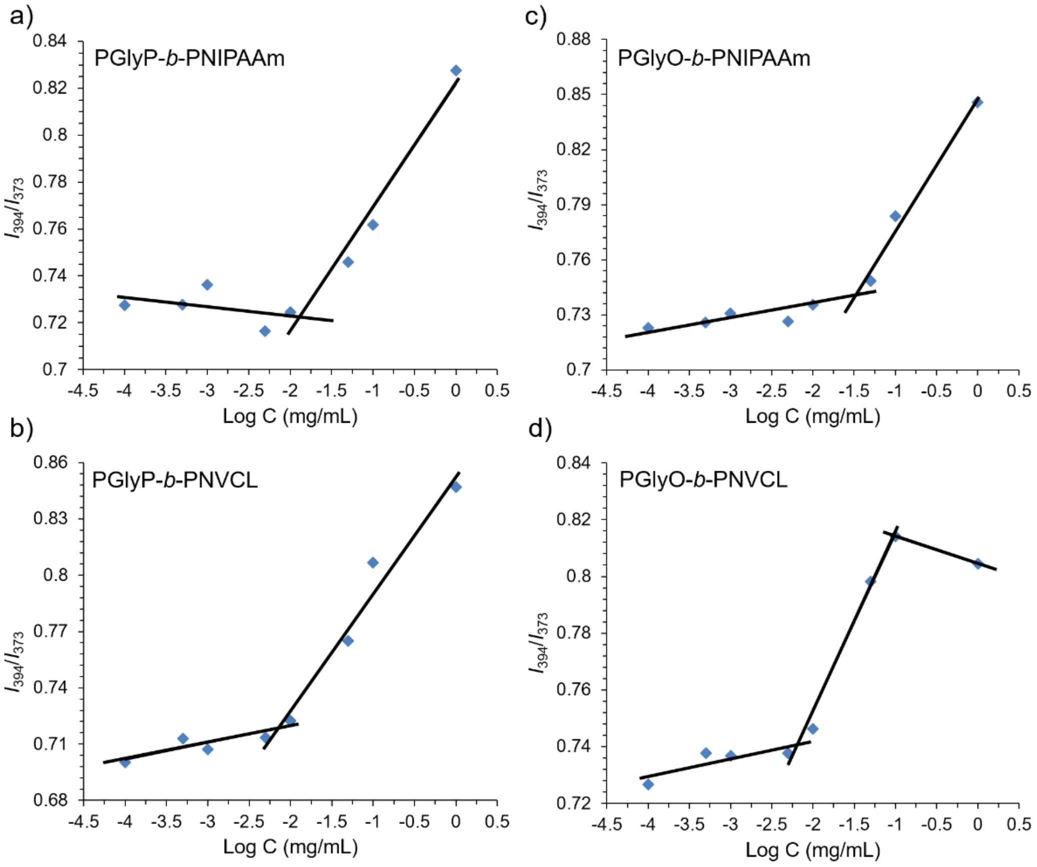

Figure S12. Intensity ratios (I_{394}/I_{373}) from pyrene emission spectra plotted with a concentration of polymers.

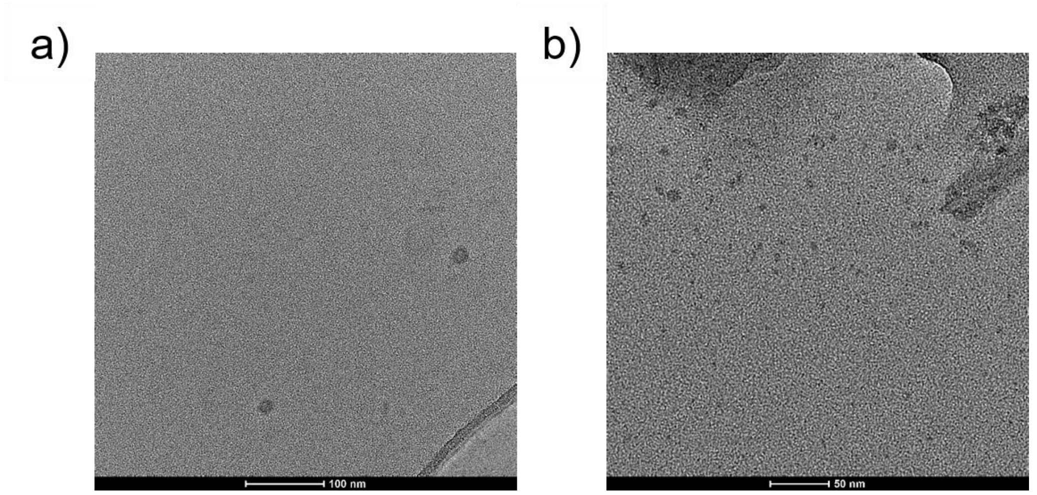

Figure S13. TEM images for a) PGlyP-*b*-PNIPAAm_NP and b) PGlyO-*b*-PNIPAAm_NP.

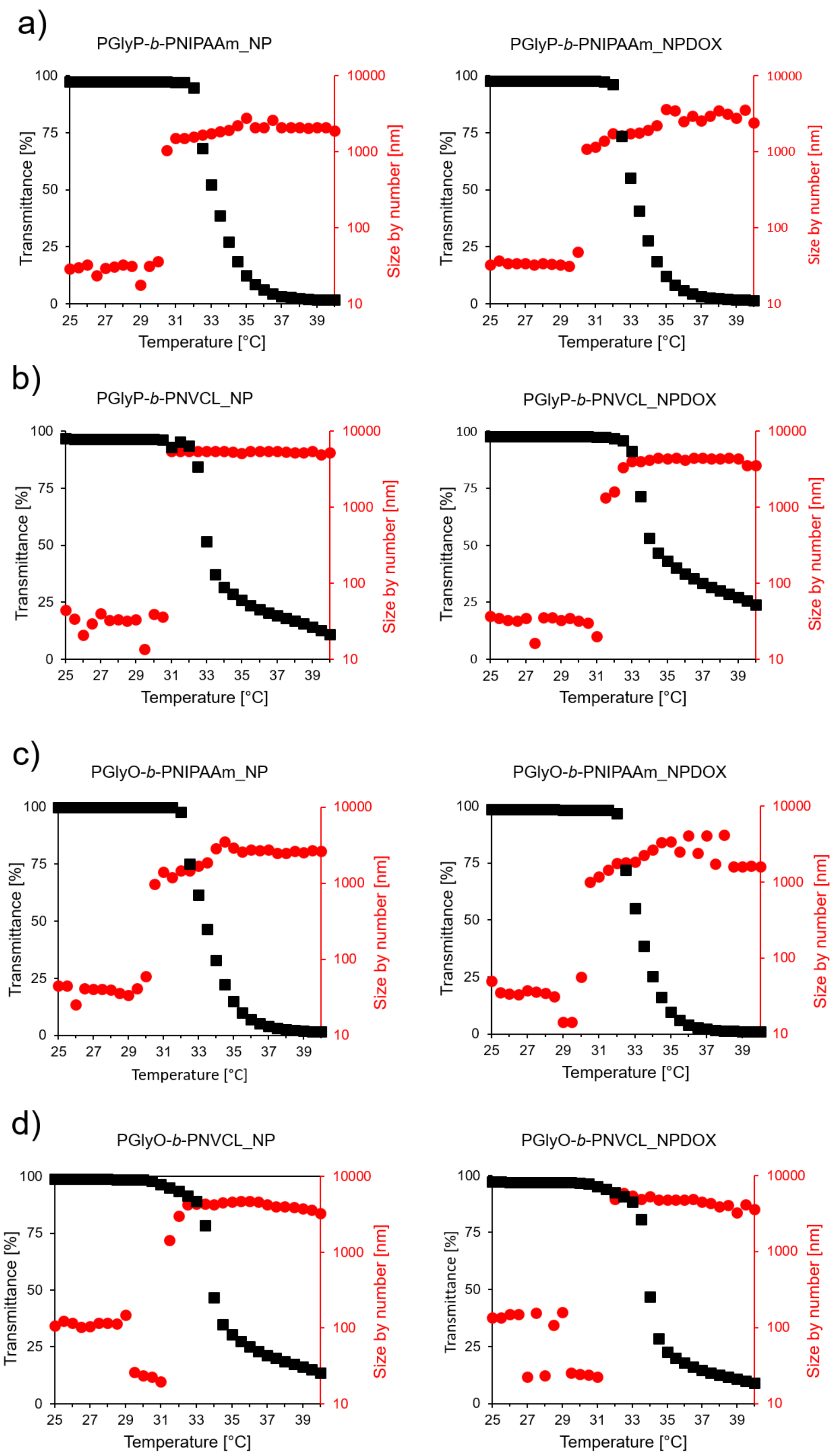

Figure S14. Evolution of transmittance measured by turbidimetry and number size distribution as a function of temperature for polymeric nanoparticles.

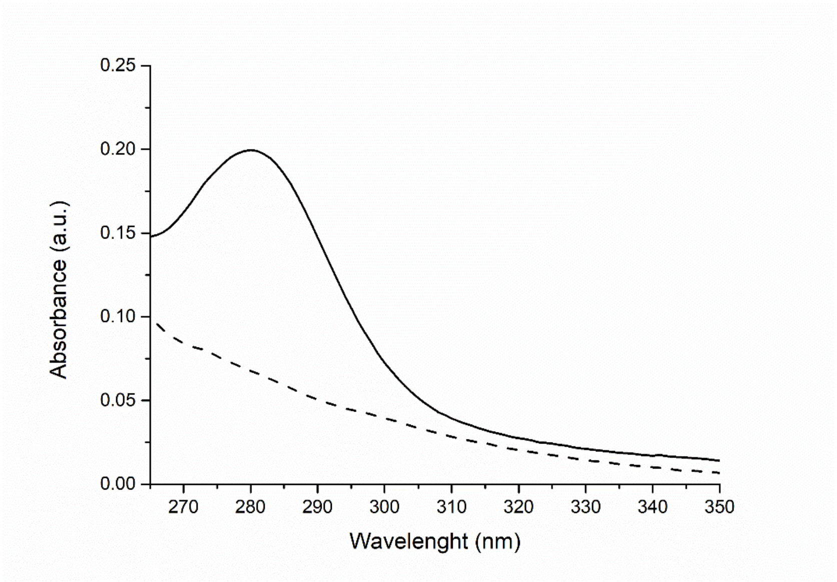

Figure S15. UV-Vis spectra of PGlyP-*b*-PNIPAAm terminated with xanthate (solid line) and reduced to thiol (dashed line).

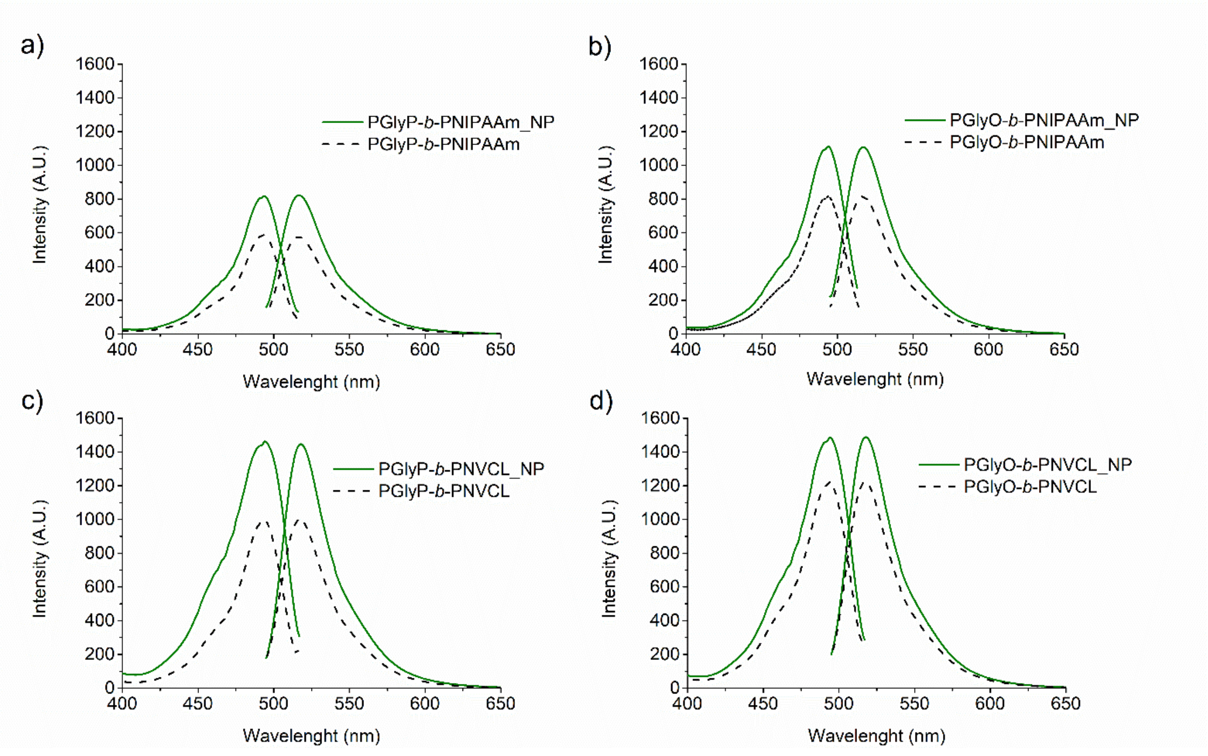

Figure S16. Fluorescence properties of polymers and corresponding polymeric nanoparticles.

Table S1. Thermal properties of the polymers.

Polymer	Maximum of the degradation rate (°C)	Residue at 600°C (%)	T_g* (°C)
**PGlyP**	400	2.0	-
**PGlyO**	405	3.3	-
**PGlyP-*b*-PNIPAAm**	415	1.8	136.5
**PGlyO-*b*-PNIPAAm**	415	2.5	135.5
**PGlyP-*b*-PNVCL**	435	1.8	174.5
**PGlyO-*b*-PNVCL**	435	2.3	165.1

Table S2. Evaluation of colloidal stability of polymeric nanoparticles.

Polymer	H_2O 24 h	H_2O 7 days	H_2O 30 days	PBS 24h	PBS 7 days	PBS 30 days
**PGlyP-*b*-PNIPAAm_NP**	34.25 ± 1.59	33.04 ± 0.38	31.36 ± 2.776	34.4 ± 1.76	30.94±1.95	31.36±0.19
**PGlyP-*b*-PNVCL_NP**	22.34 ± 1.34	34.15± 1.40	29.48 ± 0.78	27.41 ± 1.90	28.21 ± 1.89	31.61 ± 0.79
**PGlyO-*b*-PNIPAAm_NP**	42.11±0.64	39.93 ± 3.97	39.84 ± 1.74	32.59 ± 1.328	30.94 ± 0.96	37.55 ± 0.85
**PGlyO-*b*-PNVCL_NP**	24.00 ± 0.90	23.04 ± 1.31	22.34 ± 0.99	23.20 ± 1.10	23.15 ± 3.73	26.4 ± 1. 248
